# Characterisation and evaluation of the regenerative capacity of Stro-4+ enriched bone marrow mesenchymal stromal cells using bovine extracellular matrix hydrogel and a novel biocompatible melt electro-written medical-grade polycaprolactone scaffold

**DOI:** 10.1016/j.biomaterials.2020.119998

**Published:** 2020-07

**Authors:** C. Black, J.M. Kanczler, M.C. de Andrés, L.J. White, F.M. Savi, O. Bas, S. Saifzadeh, J. Henkel, A. Zannettino, S. Gronthos, M.A. Woodruff, D.W. Hutmacher, R.O.C. Oreffo

**Affiliations:** a Bone & Joint Research Group, Centre for Human Development, Stem Cells and Regeneration, Human Development & Health, Institute of Developmental Sciences, University of Southampton, SO16 6YD, UK; b School of Pharmacy, Biodiscovery Institute, University Park, University of Nottingham, Nottingham, NG7 2RD, UK; c ARC Industrial Transformation Training Centre in Additive Biomanufacturing, Queensland University of Technology (QUT), Brisbane, QLD, 4059, Australia; d Institute of Health Biomedical Innovation, Queensland University of Technology, Brisbane, QLD, 4059, Australia; e Myeloma Research Laboratory, Adelaide Medical School, Faculty of Health and Medical Sciences, The University of Adelaide, Adelaide, Australia and Precision Medicine Theme, South Australian Health and Medical Research Institute, Adelaide, Australia and Central Adelaide Local Health Network, Adelaide, South Australia, Australia; f Mesenchymal Stem Cell Laboratory, Adelaide Medical School, Faculty of Health and Medical Sciences, The University of Adelaide, Adelaide, Australia and Precision Medicine Theme, South Australian Health and Medical Research Institute, Adelaide, Australia; g College of Biomedical Engineering, China Medical University, Taichung, 40402, Taiwan; h Cartilage Epigenetics Group, Rheumatology Division, Biomedical Research Institute of A Coruña (INIBIC), Hospital Universitario de A Coruña-CHUAC, 15006 A Coruña ,Spain

**Keywords:** Stro-4, Ovine, Bone marrow mesenchymal stromal cells, Regeneration, Polycaprolactone, Extracellular matrix

## Abstract

Many skeletal tissue regenerative strategies centre around the multifunctional properties of bone marrow derived stromal cells (BMSC) or mesenchymal stem/stromal cells (MSC)/bone marrow derived skeletal stem cells (SSC). Specific identification of these particular stem cells has been inconclusive. However, enriching these heterogeneous bone marrow cell populations with characterised skeletal progenitor markers has been a contributing factor in successful skeletal bone regeneration and repair strategies. In the current studies we have isolated, characterised and enriched ovine bone marrow mesenchymal stromal cells (oBMSCs) using a specific antibody, Stro-4, examined their multipotential differentiation capacity and, in translational studies combined Stro-4+ oBMSCs with a bovine extracellular matrix (bECM) hydrogel and a biocompatible melt electro-written medical-grade polycaprolactone scaffold, and tested their bone regenerative capacity in a small *in vivo*, highly vascularised, chick chorioallantoic membrane (CAM) model and a preclinical, critical-sized ovine segmental tibial defect model.

Proliferation rates and CFU-F formation were similar between unselected and Stro-4+ oBMSCs. *Col1A1, Col2A1, mSOX-9, PPARG* gene expression were upregulated in respective osteogenic, chondrogenic and adipogenic culture conditions compared to basal conditions with no significant difference between Stro-4+ and unselected oBMSCs. In contrast, proteoglycan expression, alkaline phosphatase activity and adipogenesis were significantly upregulated in the Stro-4+ cells. Furthermore, with extended cultures, the oBMSCs had a predisposition to maintain a strong chondrogenic phenotype. In the CAM model Stro-4+ oBMSCs/bECM hydrogel was able to induce bone formation at a femur fracture site compared to bECM hydrogel and control blank defect alone. Translational studies in a critical-sized ovine tibial defect showed autograft samples contained significantly more bone, (4250.63 mm^3^, SD = 1485.57) than blank (1045.29 mm^3^, SD = 219.68) ECM-hydrogel (1152.58 mm^3^, SD = 191.95) and Stro-4+/ECM-hydrogel (1127.95 mm^3^, SD = 166.44) groups.

Stro-4+ oBMSCs demonstrated a potential to aid bone repair *in vitro* and in a small *in vivo* bone defect model using select scaffolds. However, critically, translation to a large related preclinical model demonstrated the complexities of bringing small scale reported stem-cell material therapies to a clinically relevant model and thus facilitate progression to the clinic.

## 1 Introduction

Recent advances in tissue engineering strategies *in vitro* have increased the demand for suitable *in vivo* models to progress the pre-clinical translation of candidate treatments [1]. Indeed the use and requirement for large animal models in translational medicine has been widely recognised and established over the past 20 years with canine, caprine, porcine and ovine species all used to varying degrees [[2], [3], [4]]. The use of sheep in bone tissue engineering continues to gain popularity and remains a cornerstone of orthopaedic pre-clinical research given their similarities with humans in terms of: i) weight, ii) joint structure, iii) physiology and, iv) bone structure. The increasing application of ovine models in research, therefore, increases the translational potential of the species model [5,6]. At the centre of many of the skeletal tissue regenerative strategies remains the bone marrow derived skeletal stem cell. For translational medicine, it is imperative to translate the often reported stem-cell material successes observed using small *in vitro* and *in vivo* preclinical studies to clinically relevant models at scale and thus facilitate progression to the clinic. The need to address basic questions regarding the safety and efficacy of stem-cell therapies to recapitulate bone formation and repair at scale, requires, ultimately, the use of an *in vivo* model offering physiological and biomechanical homology to humans [5]. This need has increasingly been met by the use of ovine orthopaedic models in bone tissue engineering research.

Plastic adherent ovine mesenchymal stem/stromal cells (oBMSCs) isolated from bone marrow [7,8] peripheral blood [9] and adipose tissue [10] appear fibroblastoid in culture, show similar CFU-F colony forming capacity and respond with differentiation *in vitro* and *in vivo* as the human comparator and have now been used successfully as a cell source in research utilising ovine orthopaedic models [11]. Interestingly, work to date has confirmed the expression of traditional human (mesenchymal stem/stromal cells) MSC markers on oBMSC populations including CD29, CD44, CD146 and CD166 [12,13]. However, the majority of antibodies used are not species-specific and rely on species cross-reactivity for epitope identification. Therefore, confirmation of the absence or presence of antigens must be tempered by the knowledge of the expected specificity of any antibodies used. The accepted criteria for human MSC definition include the expression of CD73, CD90 and CD105 [14,15] as markers of cell potency. In contrast, in the sheep, confirmation of CD90, CD73, CD105 and other common human MSC marker expression has not been repeatedly demonstrated, however, confirmation that reported patterns of expression are linked to species specific phenotypic difference, or merely an antibody specificity-related false negative remains challenging. Interestingly, the absence of the endothelial marker CD31 and haematopoietic marker CD45 appears shared across species. As the literature relating to the *in vitro* and *in vivo* nature of oBMSCs accumulates, it is becoming ever clearer that oBMSCs behave similarly, both *in vitro* and *in vivo*, to their human correlates. Ovine BMSCs respond to osteogenic, chondrogenic and adipogenic differentiation cues, expressing the corresponding phenotype as assessed with both histological and molecular techniques [16].

Comparable to species-specific antibody availability, oBMSCs have been successfully isolated, culture expanded, differentiated and re-implanted as both an allogenic and autogenic cell source in various applications including cardiac, maxillo-facial and bone repair research [[17], [18], [19]]. The use of immunocompetent large animal models in translational research requires the identification and characterisation of a species-specific enriched SSC population. Although candidate markers for human MSC/SSC enrichment have been detailed in the literature; including CD146, CD106, CD271, MSCA-1, CD56 and Stro-1 [[20], [21], [22]]; therapeutic targets for use in ovine pre-clinical models remain under-developed. The conventional anti-human Stro-1 antibody has been shown to bind to ovine bone marrow mononuclear cells (oBMMNCs) at a low affinity, selecting for a population 1% of the total stromal fraction [12]. This low population frequency is unattractive when selecting candidate markers for prospective cell enrichment and, to this end, Gronthos and colleagues have developed the monoclonal antibody Stro-4 as a candidate for skeletal stem cell enrichment of oBMMNCs [12]. Stro-4 designates a monoclonal antibody shown to bind specifically to human and ovine Heat Shock Protein-90. Stro-4 was produced following modification and adaption of the Stro-1 hybridoma technique, substituting the CD34 selected inoculation with a CD106 (Vascular Cell Adhesion Molecule, VCAM) BMMNC sub-population. It was later shown that the Stro-4 supernatant selected for a phenotypically separate cell subset to the parent CD106 positive cells used for hybridoma inoculation. The Stro-4 monoclonal IgG antibody has been shown to cross-react with a discrete population of human and oBMSCs. Furthermore, cells FACS selected for Stro-4 expression, were shown to enrich for CFU-F capacity some 8–16 fold, respectively [12]. Comparisons between human and ovine Stro-4+ populations demonstrated accumulation of Alizarin-Red positive deposits, alcian blue proteoglycan matrix and intracellular lipid expression following exposure to osteo/chondro and adipogenic media, *in vitro* and *in vivo*. Thus, the similarities between ovine and human MSCs appear robust, and, furthermore, Stro-4+ ovine BMSCs behave comparably *in vitro* to the human equivalent. The ability to generate cartilage, bone, stroma and marrow adipocytes is a characteristic of the skeletal stem cell, denoting an ability to form skeletal lineages, referring to the self-renewing stem cell of the bone marrow stroma responsible for the innate regenerative capacity of bone [23,24]. Thus, enriched ovine BMSCs can be regarded as a translationally relevant target for cell enrichment in ovine orthopaedic models.

In keeping with these aims of developing multifaceted solutions to augment bone formation and application in bone tissue engineering, we examined the functional characteristics of ovine bone marrow derived skeletal stem cells from cellular and molecular laboratory investigations through to evaluation in a large scale *in vivo* bone defect model. We have examined the characteristics of an oBMSC population subset selected using the Stro-4 antibody and, critically, the efficacy of the ovine enriched BMSCs for skeletal repair assessed using a bone extracellular matrix hydrogel and melt electro-written medical-grade polycaprolactone (mPCL) tubular scaffolds. Regeneration was examined in a highly vascularised small *in vivo* model as well as in a large clinically relevant segmental ovine bone defect model.

## 2 Material & methods

### 2.1 Materials

The Stro-4 antibody was generated as previously detailed by Zannettino and colleagues, University of Adelaide [12]. Foetal calf serum was obtained from Life technologies, Scotland UK. Remaining cell culture reagents were purchased from Sigma-Aldrich, UK unless stated. Fertilised eggs were purchased from Medeggs, Norfolk, UK.

#### 2.1.1 Stro-4 magnetic activated cell separation of oBMSCs

Ovine Stro-4 cell isolation from whole bone marrow was adapted from protocols to isolate human Stro-1 from human bone marrow aspirates [25,26]. Briefly, 5–8 mL of sheep iliac crest bone marrow aspirate was obtained aseptically immediately post-mortem. Marrow samples were collected in basal tissue culture medium (alpha-MEM, 10% foetal calf serum, penicillin/streptomycin) washed and filtered into single cell preparations before undergoing density dependant centrifugation, erythrocyte depletion and mononuclear cell fraction enrichment. The enriched mononuclear fraction was prepared for antibody labelling by incubation in a protein blocking solution containing 2% human and bovine serum. Cells were then centrifuged and re-suspended, washed with 1x PBS and approximately 1 × 10^8^ cells were incubated in neat Stro-4 supernatant on ice for 30 min. Cells were washed in 1x PBS, and the Stro-4 labelled oBMSCs were incubated with goat anti-mouse IgG MicroBeads (Miltenyi Biotech, UK) at 20 μL per 1 × 10^7^ cells (approximately 200 μL of beads) for 15 min on ice. The labelled cells were then re-suspended in MACS buffer, passed through a MACS column magnets (QuadroMACS and MACS columns, Miltenyi Biotech), and the positive fraction from each sample was collected, counted and seeded onto standard tissue culture plastic cell culture flask at 1× 10^4^ cells per cm^2^. Cells were cultured in basal tissue culture medium at 37 °C, 5% CO_2_ in balanced air.

### 2.2 Flow cytometric analysis

Cultured ovine MSCs were incubated in blocking buffer (Hanks Buffered Saline Solution + 20 mM Hepes, 1% normal human AB serum, 1% bovine serum albumin (BSA: Cohn fraction V, Sigma Aldrich Pty Ltd, NSW, Australia), and 5% FCS for 20 min on ice. For each analysis, 1 × 10^5^ cells were resuspended in 100 μL of 10 μg/mL primary antibody, anti-CD14, -CD31, -CD34, -CD45 (BIO-RAD Hercules, CA), anti-STRO-4, -CD29, -CD44 [12], anti-CD90, or –CD166 (BD Biosciences San Jose, CA) for 45 min on ice. The isotype-matched, non-binding control antibodies, antibodies, IgG (BIO-RAD) was used as culture supernatant under identical conditions. The cells were then washed in HBSS with 5% FCS and incubated with a goat anti-mouse IgG (γ-chain specific) phycoerythrin (PE) (1/50; Southern Biotechnology Associates, Birmingham AL) for 30 min on ice. Prior to analysis, cells were washed twice in HBSS with 5% FCS and resuspended in PBS/1% paraformaldehyde. Flow cytometric analysis was performed using an Epics-XL/MCL flow cytometer (Beckman Coulter, Hialeah, FL). The analysis was performed using the FlowExpress software.

### 2.3 Proliferation and Colony Forming Unit capacity of Stro-4+ vs unselected oBMSCs

#### 2.3.1 Population doubling time

Population doubling time was measured by plating 1 × 10^3^ cells/cm^2^ in T75 flasks, growing cells in basal media until approximately 60% confluence. Cells were washed in 1x PBS, enzymatically digested (1x Trypsin/EDTA solution), washed and centrifuged before resuspension in 2 mL of culture media. The total number of cells was recorded before cells were re-plated at 1 × 10^3^ cells/cm^2^. This process was repeated from passages 0–5 using a total of 5 animals. Population doubling time, the time taken in hours for the total number of cultured cells to double in number, was calculated as follows:

Population Doubling Time = Time (in Hours) Log2 [Log (Final Cell Number) – Log (Seeded Cell Number)]

#### 2.3.2 Colony Forming Unit capacity (CFU–F)

Assessment of clonogenic capacity was carried out using the Colony Forming Unit-Fibroblast (CFU–F) assay [27]. Cultured Stro-4+ and unselected cells from early passage 0 to late passage 5 (1 × 10^1^/cm^2^), were plated into T-25 flasks and cultured in basal culture medium. 14 day cell cultures were washed in 1x PBS, fixed in 85% ethanol before being air-dried. The fixed cultures were stained for alkaline phosphatase (ALP) by incubation with naphthol AS-MX phosphate (40 μL/mL) and Fast Violet B salt (2.5 μg/mL) in distilled H_2_O for 45 min at 37 °C under 5% CO_2_ in a humidified atmosphere in the dark. ALP^+ve^ colonies (colonies exhibiting ≥50% ALP^+ve^ stain across their diameter) were labelled and counterstained for 5 min with haematoxylin. Colonies comprising of >32 cells were analysed. Colonies were washed in 1x PBS air dried, imaged and counted via light microscopy.

### 2.4 Multi-lineage differentiation of oBMSCs

#### 2.4.1 2-Dimensional *in vitro* culture assay

The potential for Stro-4+ oBMSCs to differentiate into osteogenic, chondrogenic and adipogenic lineages was carried out as previously stated for human skeletal stem cells [28] with adaptations outlined briefly due to the accelerated growth characteristics observed for ovine cells. Differentiation of the cells into the three lineages was assessed at day 21 using both histological and molecular techniques of cells grown in monolayer culture.

Each donor sample was plated in a 6 well plate at 2 × 10^4^ cells per well, two wells per condition, both Stro-4+ and unselected cells. Two wells per condition for both cell types were cultured in basal media as experimental controls. Cells were first cultured in basal media; 10% FCS, 1% P/S, α-MEM until 20–30% confluent then switched to 1.5 mL per well lineage-specific media (**Osteogenic**: α-MEM with 10% FCS, 100 μM Ascorbate-2-phosphate, 10 nM Dexamethasone, 25 nM 1α, 25-dihydroxy Vitamin D3, 10 mM β-glycerophosphate. **Chondrogenic:** α-MEM with 100 μM Ascorbate-2-phosphate, 10 nM Dexamethasone, 1% insulin, transferrin, selenium (ITS) medium supplement, and 10 ng/mL TGF- β3. **Adipogenic:** α-MEM with 3 g/l D + Glucose, 10% FCS, 3 μg/mL insulin, transferrin, selenium (ITS), 100 nM Dexamethasone, 0.5 mM IBMX, and 1 μM Rosiglitazone). Culture media was changed twice a week over 21 days. Cells were either fixed appropriately for histological staining or lysed in preparation for molecular analysis.

#### 2.4.2 Chondrogenic, osteogenic and adipogenic staining of unselected and STRO-4+ enriched oBMSCs

The monolayer cell cultures were washed twice with PBS and then fixed with 4% paraformaldehyde at room temperature for 15 min. Another series of washes were done with Mili-Q H_2_O (each wash at 5 min). For chondrogenesis; cells were stained with 0.5% Alcian blue 8GX for proteoglycan-rich cartilage matrix for 15 min and washed with 1x PBS. For osteogenesis; 0.2 mL of 40 mM alizarin red solution was added and incubated for 1 h on a rotating plate. The samples were then washed 5 times with dH_2_O. Alizarin red was used to stain calcium deposits with orange/red colour. For adipogenesis, Oil Red O staining was used. Cell cultures were fixed in Baker's formal calcium, washed in 60% isopropanol, and stained with double-filtered Oil Red O solution for 15 min to show for cytoplasmic lipid accumulation. All stained cells were imaged under using a Carl Zeiss Axiovert 200 microscope. Carl Zeiss Axiovision 3.1 software package was used to capture the stained cells.

#### 2.4.3 Stro-4+ vs unselected *in vitro* characterisation

Unselected and Stro-4+ cells were culture expanded in basal tissue culture medium at 37 °C, 5% CO_2_ in balanced air. After the first passage, cells were seeded into 6-well plates at 2 × 10^4^ cells per well, three well per condition (and with 3 replicates, 9 wells per condition), and grown in basal media. As previously stated cell culture media was switched to chondrogenic or osteogenic as appropriate and culture extended to 14 days with media changed every 72 h. Cells were fixed and stained with Alcian Blue (chondrogenic differentiation) and Alizarin Red (osteogenic differentiation) as previously stated and quantified for staining intensity following an automated pipeline using an open source image analysis software (CellProfilerTM, The Broad Institute, USA). Three x4 magnification images were captured across the equator of the well. Images were uploaded into CellProfiler and analysed using the pipeline: convert to greyscale > Area of interest colour red > Quantify (Supplementary fig.1) Overall intensity was normalised to the cell count per image field by performing an object count on DAPI stained images. Staining intensity of cells cultured in basal, chondrogenic or osteogenic media was expressed as intensity units per cell.

#### 2.4.4 3-Dimensional chondrogenic *in vitro* culture assay

Ovine Stro-4+ and unselected cells were grown in micromass cultures. In brief, passage 1 cells were resuspended in basal media at a concentration of 1 × 10^4^/μL. Micromass cultures were formed by depositing 30 μL of cell suspension into a well of a 6-well plate, droplets were pipetted carefully on the plate surface. Three micromass cultures were placed per well then allowed to adhere by incubating for 2 h at 37 °C. Cultures were gently covered with 1.5 mL standard chondrogenic media and cultured for 21 days.

#### 2.4.5 Molecular analysis

Unselected and Stro-4+ skeletal populations obtained from different sheep (n = 4) were cultured for 21 days in conditioned media for tri-lineage differentiation, as previously stated. The potential to differentiate into adipocytes, osteoblasts and chondrocytes was assessed using relative gene expression levels of differentiation markers using quantitative RT-PCR (Supplementary Table 1). Total RNA (500 ng) was extracted from cultured samples using an Isolate II RNA Mini kit (Bioline), according to the manufacturer's instructions. RNA was immediately reverse-transcribed with TaqMan Reverse Transcription Reagents (Applied Biosystems). RNA concentration was evaluated using a NanoDrop 1000 spectrophotometer (Thermo Scientific, UK). Relative quantification of gene expression was performed with an ABI Prism 7500 detection system (Applied Biosystems). Primer Express 3.0 software (Applied Biosystems) was used to design all primers in the current study. The 20-μL reaction mixture was prepared in triplicate, containing 1 μL of complementary DNA, 10 μL of GoTaq qPCR Master Mix (Promega), and 1 μM of each primer. Thermal cycler conditions included an initial activation step at 95 °C for 10 min, followed by a 2-step PCR program of 95 °C for 15 s and 60 °C for 60 s for 40 cycles. The 2^−ΔΔCt^ method was used for relative quantification of gene expression, and the data were normalised to β-actin expression (Supplementary Table 1 – Primer sequences).

### 2.5 *In vivo* assessment of Stro-4+ oBMSCs in bone regeneration

#### 2.5.1 Ex-vivo femur defect cultures in the chorio-allantoic membrane model (CAM)

Chick femurs were harvested from embryonic day 18 chicks by carefully removing the proximal and distal hind limbs. The femur was carefully disarticulated distally from the femoro-tibial joint, and proximally at the femoro-acetabulum. Precaution was taken to ensure that neither cartilaginous epiphysis were removed from the calcified diaphysis. Once the femora were isolated, all remaining extra-osteal tissue was removed. Freshly isolated femora were placed in α-MEM and kept overnight in a tissue culture incubator (37 °C, 5% CO_2_ in balanced air).

All egg CAM procedures were carried out in accordance with the guidelines and regulations stipulated in the Animals (Scientific Procedures) Act, UK 1986. The chick embryo CAM model was under Home Office Project license (PPL 30/2762). On the day of implantation into the CAM, sequentially; each femur was removed from storage media and placed onto sterile filter paper to remove excess culture media, then using a scalpel, the femur was transected mid-shaft. An in house-built melt eletrowriting device, a technology combining additive manufacturing and electrospinning system, was used to fabricate the tubular mPCL scaffolds with medical-grade polycaprolactone (Purasorb PC 12, Purac Biomaterials, The Netherlands) following a previously described methodology [29]. Next, sections of sterile melt electro-written mPCL tubular scaffolds were cut, approximately 5 mm in length and 2 mm in diameter. Using fine-toothed forceps, the mPCL scaffold was carefully pulled over the sectioned ends of each femoral half attempting to leave a 2 mm gap or defect between femoral segments (Supplementary Fig. 2).

Once each femur was secured in place using a 5 mm scaffold segment, the femur/scaffolds were placed in a sterile dish and held in place by forceps. Femurs were divided according to experimental groups. Group I: Femur and mPCL scaffold (n = 6); Group II: Femur/mPCL scaffold/ECM hydrogel (n = 6); Group III: Femur/mPCL scaffold/ECM hydrogel and Stro-4+ oBMSCs (n = 6). Bovine bone extracellular matrix (bECM) hydrogel was prepared using a protocol adapted from Pietrzak et al., 2011 [30] and further characterised by Sawkins et al.*,* 2013 [31]. In brief, bovine bone samples underwent sequential morsalisation, demineralisation to form a demineralised bone matrix (bDBM), chloroform/ethanol-based lipid removal and a final decellularisation step to produce a bovine bECM material. bECM was subjected to solubilisation and pepsin digestion to prepare a functional hydrogel [30,31]. In groups containing the hydrogel, the needle was inserted into the lumen of the scaffold and femoral defects with care taken not to damage any construct components nor dislodge either end of the femur. Once the constructs were completed, they were placed onto the CAM. CAM eggs with implants were sealed and placed into the Hatchmaster (Brinsea UK) with rotation turned off and cultured for 8 days.

#### 2.5.2 Ovine tibial segmental defect model

The studies were undertaken following approval from the animal ethics committee of the Queensland University of Technology (animal ethics approval no. 16). Sixteen experimental animals (aged male, > 6 years old, merino-cross sheep) were divided amongst two experimental groups, (Supplementary Table 2).

For the sheep tibial defect model, tubular mPCL scaffolds with a length of ~6 cm and diameter of ~2 cm were fabricated using melt electrowriting technology (Supplementary Fig 3). bECM hydrogel and mPCL scaffold constructs without cells (n = 8) and bECM hydrogel and mPCL scaffold constructs seeded with Stro-4+ oBMSCs (n = 8) (Supplementary Fig. 2). Surgical protocols were based on those published by Reichert et al,. 2010 [32]. In brief, a linear incision, approximately 12 cm in length was made on the medial aspect of the distal pelvic limb, extending proximally from a point 1 cm below the tibial plateaux and distally to the medial malleolus. A customised dynamic compression plate (DCP), 5.2 mm broad, 10 holes, (Synthes™), was contoured to the morphology of the bone with a plate bending press (Synthes™) and the fit confirmed against the intact bone. The distal end of all plates was placed exactly 2.5 cm proximal of the medial malleolus.

A line marking the centre of the defect was made using a rasp. A point 1.5 cm proximal and 1.5 cm distal to the centre mark was made using the oscillating saw. These defined precise osteotomy lines for a 3 cm tibial diaphyseal defect. Screw holes were pre-drilled, 3 proximally and 2 distally to the osteotomy line. The periosteum was removed above and below the defect to inhibit the healing response and maximise the regenerative challenge. The tibial diaphyseal defect was created with limb stability provided by a customised unilateral 5.3 mm DCP secured with 3.5 mm self-tapping screws.

Melt electro-written scaffolds were applied, sutured in place and anchored by application of the DCP plate on the abaxial surface of the defect. In both groups, 8 mL of sterile bovine bECM pre-gel solution was injected into the defect lumen, localised by mPCL scaffold. Seeded bECM pre-gel solution were combined with cultured ovine Stro-4+ oBMSCs at a density of 4 × 10^6^/mL of gel material. Seeded and unseeded treatments were warmed to room temperature immediately prior to application and gelation allowed to occur *in situ* before the defect wound was sutured closed (Supplementary Fig. 4).

The operated limb was cast in fibreglass tape which was split after one week and used as a bivalve splint for a further two weeks. The animals were not immobilised following surgery and were permitted to weight-bear immediate following recovery from surgery. The full extent of bone weight-bearing was minimised during the first 3 weeks post-operation by the leg cast and splint. Animals were routinely examined for lameness, inflammation, infection and wound complication. All animals recovered without complication from surgery. Two animals were removed from the study after 6 weeks due to plate bending, no animals were excluded relating to complications of bone fracture. The remaining fourteen animals continued through to 3 months without clinical complications. Findings were combined with historical data from empty and autograft controls.

### 2.6 Analysis of *in vivo* models

#### 2.6.1 Micro-computed tomography (μCT)

3D analysis of PFA fixed organotypic cultured chick femurs/hydrogels, and chick femur defects/hydrogels with Stro-4+ cells were performed using a SkyScan 1176 scanning system (Bruker μCT, Kontich). Samples were scanned at 18 μm resolution and reconstructed using NRecon software interface (v.1.6.4.6, Bruker μCT, Kontich). Reconstructed femurs and hydrogels were analysed using CT Analyser (v.1.13.2.1+, Bruker μCT, Kontich) and images generated using CT Vox 3.0.

For the ovine tibial segmental defect model, fixed bone samples were scanned at 18 μm resolution using a Scanco μCT 40 micro-CT, files were converted to .ISQ format and analysed in combination with control data at the University of Southampton using ctAn software (Bruker μCT, Kontich). The defect region was identified using the drill holes nearest to the defect boundary as landmarks, with the defect universally defined 0.5 cm proximal and distal to the respective drill holes. Thresholding was set uniformly for all analysis, advised on an Otsu analysis. The defect region was analysed in four segments, proximal, middle, distal and whole for bone volume (BV mm^3^). Only values for the whole defect region were included due to the absence of new bone formation demonstrated in test groups. Bone volume was calculated using Bruker Skyscan® ctAn software.

#### 2.6.2 Chick femur/CAM histology

Following μCT analysis, chick femur defect samples were dehydrated through a series of ethanol washes (50%, 90% and 100% in dH_2_O) and incubated in Histo-Clear (National Diagnostics). Following incubation in paraffin wax for 1 h at 60 °C, samples were embedded in wax blocks using an automated Shandon Citadel 2000. Consecutive 7 μm thick sections were cut throughout the depth of the central hydrogel insert. Mounted sections were rehydrated through Histo-Clear, graded ethanol's and dH_2_O before staining for the nuclear counter-stain Weigert's haematoxylin, followed by staining with 0.5% Alcian blue 8GX for proteoglycan-rich cartilage matrix and 1% Sirius red F3B for collagenous matrix. Additionally, separate slide sections were stained for Goldner's Trichrome to detect bone and osteoid according to standard protocols. Sections were then dehydrated and mounted with DPX before imaging with an Olympus BX-51/22 DotSlide digital virtual microscope using OlyVIA 2.1 software (Olympus Soft Imaging Solutions, GmBH).

#### 2.6.3 Sheep tibial defect histology

Following μCT analyses, tibial bone specimens were frozen and trimmed to 6 cm length and fixed in freshly made 4% paraformaldehyde at 4 °C for a minimum of one week, with fixative replaced every 72 h. For histological analysis, the mid-defect regions were sectioned in the transverse and sagittal plane. Sections were split between resin and paraffin embedding. Paraffin-embedded sections were used for haematoxylin and eosin (H&E) staining. Samples for paraffin embedding were first decalcified in 15% EDTA for 6–8 weeks at 4 °C with regular EDTA changes. Decalcified samples were dehydrated by sequential rising percentages of ethanol using an automated tissue processor (Excelsior ES, Thermo Scientific, Franklin, MA, USA), and embedded in paraffin wax. Samples were sectioned (5 μm) using a microtome (Leica RM 2265). The slides were deparaffinised with xylene and rehydrated prior to H&E staining (Sigma Aldrich). Samples were mounted with Eukitt (Fluka Biochemica, Milwaukee, WI, USA).

Samples which were resin embedded were fixed in 4% paraformaldehyde, and serially dehydrated in ethanol from 70% to 100% graded ethanol for approximately one week in each solution. The samples were then degreased in xylenes for 8 h.

Following degreasing with xylene, samples were infiltrated and embedded in the low-temperature embedding system Technovit 9100 New® (Heraeus Kulzer GmbH, Germany) according to Wilbold et al., 2010 [33]. After polymerisation, the resin blocks were mounted and sectioned longitudinally at 200 μm using an EXAKT 310 Diamond Band Saw and subsequently ground at 60 and 70 μm using an EXAKT 400CS micro grinder (EXAKT Apparatebau GmbH & Co. KG, Norderstedt, Germany) according to the technique described by Donrath., 1995. [34]. Histological staining was performed using Goldner's trichrome staining according to previously published methods [35].

## 3 Statistical analysis

Statistical analysis of comparisons in cellular staining intensity were compared by ANOVA with multiple comparisons between groups assessed by Tukey's post hoc multiple comparison test using GraphPad Prism 6 software version 6.0. Statistical analysis of gene expression was performed by ANOVA, with LSD non-parametric post hoc testing for intergroup comparison using SPSS software, expression was normalised relative to unselected population in basal media. Graphs were generated using GraphPad Prism. For the Sheep study, statistical analysis was performed using GraphPad Prism 6 software version 6.0 with graphs produced in the same software. A one-way analysis of variance ANOVA was used with Tukey's post-hoc test comparing the means of each group with one another, and Dunnett's post hoc comparing each group to blank controls. Significance at p < 0.05 was considered significant. Only autograft demonstrated significant bone growth, ****p < 0.0001.

## 4 Results

### 4.1 The expression of Stro-4 in OBMSCs

**Fig. 1:**
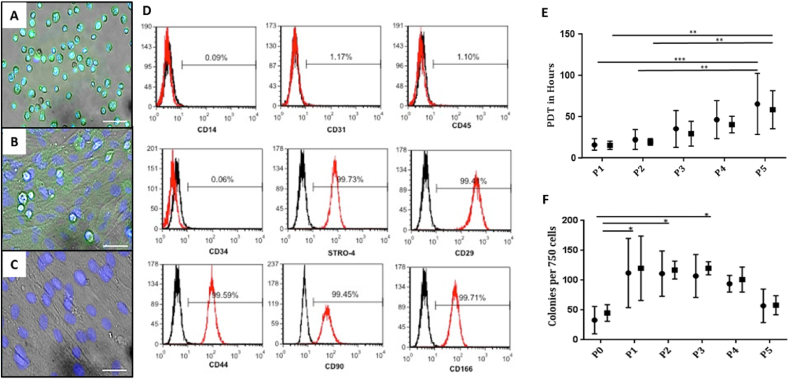
**Fluorescence microscopy demonstrating Stro-4 expression in oBMSCs (A**–**C).** Stro-4 (green), nuclear counter stained blue (DAPI). Cells in suspension immediately post MACS isolation of fresh ovine bone marrow mononuclear cells (A). Stro-4 expression of adherent passage 0 cells (B). Loss of Stro-4 expression by the end of passage 3 (C). Scale bar = 50 μm. **Immunophenotype of cultured sheep MSC (D)**. Single cell suspensions of passage 2 ovine MSC were assessed for their expression of cell surface levels of MSC associated markers (STRO-4, CD29, CD44, CD90, CD166), haematopoietic markers (CD14, CD34, CD45) and endothelial marker (CD31) by flow cytometric analysis. The horizontal line was set to the reactivity levels of <1.0% mean fluorescence obtained with the isotype-matched control antibody (black line) treated under the same conditions. The histogram represents 2 × 10^4^ events **(D). Population Doubling Time (PDT, in hours) of oBMSCs cultured in vitro (E)**. Comparative growth rate of Stro-4 (■) and Unselected oBMSCs (●). Error bars = mean ± standard deviation, N = 5 for all time points and both cell groups. **p < 0.01; ***p < 0.001. **Stro-4 and Unselected oBMSCs Colony Forming Unit capacity with passage** (**F**). Stro-4 (■) and Unselected oBMSCs (●) displayed similar colony--forming capacity from early to late passage. P0 cells demonstrated poor colony-forming capacity in comparison to that seen at P1 and sustained until P3, slowly tapering off at late passage (P5). Error bars = mean ± standard deviation, N = 5, *p < 0.05. (For interpretation of the references to colour in this figure legend, the reader is referred to the Web version of this article.)

The presence of Stro-4 antigen expression was confirmed by fluorescent immunolabeling of freshly sorted cells with temporal expression of Stro-4 monitored by immunofluorescence over successive passages until a signal was no longer detectable (Fig. 1A–C). The expression of Stro-4 declined rapidly after initial passage and was uniformly absent in passage 3 cells. The absence of Stro-4 at passage 3 subsequently determined the definition of early and late passage cells (presence and absence of Stro-4 antigen). Furthermore, increased subject samples, tested at P0, showed a significant increase in CFU-F population numbers in the Stro-4+ enriched populations compared to the unselected cells (Supplementary Fig. 5).

### 4.2 *In vitro* characterisation of Stro-4+ skeletal cell populations

Single cell suspensions of passage 2 ovine skeletal populations were assessed for their expression of cell surface levels of: i) MSC associated markers (STRO-4, CD29, CD44, CD90, CD166), ii) haematopoietic markers (CD14, CD34, CD45) and iii) endothelial marker (CD31) by flow cytometric analysis. Ovine BMSCs expressed high levels of MSCs associated markers – specifically CD29 (99.4%), CD44 (99.59%), CD90 (99.45%), CD166 (99.71%) and Stro-4 (99.73%) (Fig. 1D). Negligible expression of the endothelial associated adhesion marker CD31 (1.17%) and the haematopoietic markers CD45 (1.10%) and CD34 (0.06%) was observed.

### 4.3 *In vitro* growth characteristics -*population doubling time (PDT)*

Skeletal stem cell primary culture is characterised by a variable period of adhesion enabling cell recovery, expression of adhesion molecules, settling and adherence to tissue culture plastic. Initial population doubling times (PDT) were observed to be 100 cells per cm^2^ at initial seeding. The population doubling time remained at between 20 and 30 h for passages 1 and 2 with growth rate declining from passage 3 with a PDT of 24–64 h. The growth profile for selected and unselected cells, over passages 4 and 5, reduced to give a PDT range between 38 and 110 h at P5, (Fig. 1E). Variation between samples and across both groups was noted to increase with passage. The observed variations in growth profile correlated with changes in morphology and CFU-F capacity over time and passage (Fig. 1E).

### 4.4 Colony Forming Unit-Fibroblast (CFU–F) assay

CFU-F potential was assayed by contact independent tissue culture plastic adherence and proliferation with selected and unselected populations indistinguishable, statistically, at each measured CFU-F time point. CFU-F numbers were observed to be lowest for all passages at 39 ± 9 colonies/750 cells. Primary passage cell growth was significantly lower than P1 and P2 in unselected and Stro-4+ populations, while, CFU-F numbers in selected populations, at P3, was significantly higher than unselected primary cultures. CFU-F numbers were noted to be consistent at 102 ± 8 colonies/750 cells between passages 1–4 for both cells groups over all time points. Colony formation was noted to decline at passage 5 in selected and unselected populations but did not reach statistical significance (Fig. 1F).

## 5 Tri-lineage differentiation of ovine Unselected and Stro-4+ oBMSCs at early and late stage passage culture

**Fig. 2:**
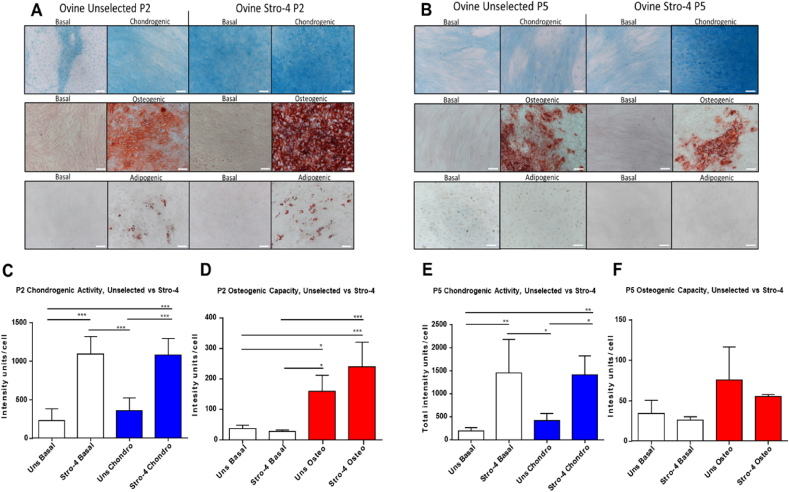
**Tri-lineage differentiation of passage 2 (A) & passage 5 (B) Unselected and Stro-4 enriched ovine BMSCs and quantification of chondrogenic (Alcian Blue) and osteogenic (Alizarin Red) staining in passage 2 (C & D) and passage 5 (E & F) Unselected (Uns) and Stro-4 populations**. An enhanced osteogenic response is seen in the Stro-4 population over unselected, Alizarin red stain (A). Chondrogenic response is comparable between Stro-4 and unselected cell groups with inherent chondrogenic activity noted in the Stro-4 population under basal conditions, Alcian blue stain (A). Adipogenic response in both groups was present but unconvincing, assessed by Oil Red O staining (A). Passage 5 cells response to osteogenic media was present in both cell groups although diminished compared to passage 2, Alizarin red (B). The chondrogenic response in unselected cells had diminished at passage 5 but was conserved in the stro-4 enriched population, Alcian blue (B). Adipogenic differentiation is absent in both populations. N = 4, both groups for each condition. Scale bar = 200 μm P2 Stro-4 enriched oBMSCs showed increased alcian blue staining intensity compared to Unselected oBMSCs Stro-4 enriched cells (C). Both P2 populations demonstrated a positive phenotypic change under osteogenic conditions but did not show a significant difference between unselected and enriched populations (D). In contrast to chondrogenic differentiation, no innate osteogenic response was noted under basal culture conditions (d). Under both basal and chondrogenic conditions, P5 Stro-4 enriched oBMSCs responded positively showing significant increases in staining intensity compared to Unselected cells (E,F). The response pattern between groups appears similar to that seen at passage 2 (E,F). Cellular response to osteogenic media diminished in late passage (F). Although an osteogenic response was present there was no significant difference in osteogenic response between conditioned and basal media (F). N = 4, error bar = mean ± SD. . *p < 0.05, **p < 0.005, ***p < 0.001. (For interpretation of the references to colour in this figure legend, the reader is referred to the Web version of this article.)

Early and late passage Stro-4+ selected and unselected oBMSCs were observed to grow rapidly under basal culture conditions. Early passage Stro-4 enriched populations displayed lineage-specific differentiation under basal and chondrogenic conditions compared to unselected cells (Fig. 2A,C). A similar response was observed in late passage 5 cells (Fig. 2B,E). Under osteogenic and adipogenic culture conditions differentiation was observed in Stro-4+ and unselected populations at early passage (Fig. 2A,D) compared to basal cultured cells. Minimal osteogenic and adipogenic differentiation was observed in late passage (P5) cells (Fig. 2B,F).

## 6 Molecular analysis of osteogenic-, adipogenic- and chondrogenic gene expression in un-selected and selected Stro-4+ oBMSCs

**Fig. 3:**
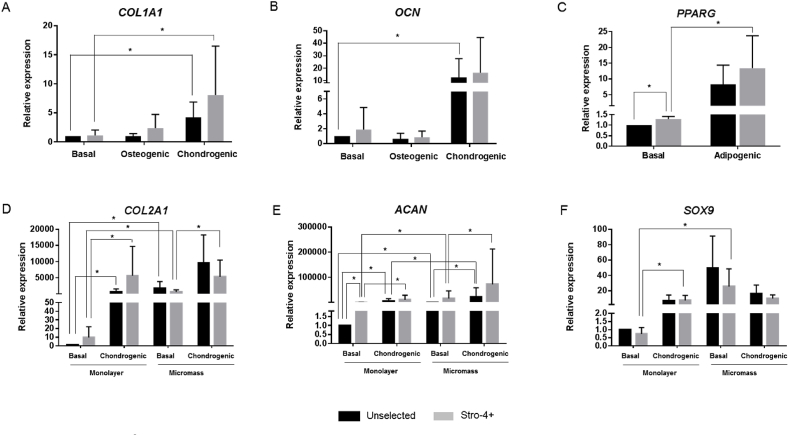
**Relative expression of differentiation markers of unselected and selected (Stro-4+) cells in monolayer and micromass cultures for 21 days**. Expression of osteogenic (A–B), adipogenic (C) and chondrogenic (D–F) markers was analysed by quantitative reverse transcription-polymerase chain reaction. Data are shown as median ± SD of triplicate determinations per sample (n = 4). Statistical differences were calculated by Mann-Whitney *U* test (*P < 0.05).

Expression of the osteogenic markers *COL1A1* and osteocalcin (*OCN*) was observed to be variable between media conditions and cell populations. Stro-4+ cell cultures showed a 1.64-fold increase under osteogenic conditions, while no increase in *COL1A1* gene expression was observed for unselected cells (Fig. 3A). Interestingly, *COL1A1* gene expression was upregulated 4.38-fold increase in unselected and 5.36-fold increase in Stro-4+ cells across both cell populations under chondrogenic culture conditions (Fig. 3A). No significant differences were found in *OCN* expression in both cell populations cultured in osteogenic media conditions (Fig. 3B). However, under chondrogenic conditions, potential upregulation of *OCN* was observed with unselected cells displaying a 6.59-fold increase and Stro-4+ cell cultures 3.04-fold increase compared to basal cultured cells (Fig. 3B).

*PPARG* mRNA expression levels were modestly increased, under basal conditions Stro-4+ cells showed a 1.27-fold compared with unselected cells. *PPARG* expression was significantly increased under adipogenic conditions, showing in unselected cells 3.34-fold increase and, a 4.07-fold increase in Stro-4 selected populations, although these did not reach statistical significance (Fig. 3C).

The chondrogenic markers *COL2A1*, aggrecan (*ACAN*) and *SOX9* showed significant gene expression increase under chondrogenic culture conditions in both unselected and Stro-4+ cells (Fig. 3D–F). Unselected cells showed a 216-fold increase in *COL2A1* mRNA levels whilst Stro-4+ cells demonstrated a 1760-fold increase in chondrogenic conditions compared to basal conditions in monolayer culture. Stro-4+ populations also showed a 6.12-fold increase in *COL2A1* gene expression when cultured in basal conditions compared to unselected cells (Fig. 3D). A similar response with *ACAN* gene expression was observed, unselected cells showed a 630.34-fold increase compared to a 1831.34-fold increase in the Stro-4+ cells under chondrogenic conditions. Moreover, *ACAN* gene expression showed a 20.44-fold increase in Stro-4+ cells under basal conditions compared to unselected cells (Fig. 3E). *SOX9* expression levels were upregulated in chondrogenic culture conditions, showing a 5.98-fold increase in unselected cells compared to 6.34-fold increase in Stro-4 populations (Fig. 3F).

3D micromass cultures using unselected and selected populations showed a significant increase compared to the basal cultured groups. *COL2A1* relative expression showed a 1681.9-fold increase and a 415.7-fold increase in unselected and Stro-4+ populations, respectively (Fig. 3D). For *ACAN* relative expression levels, Stro-4+ cells showed a 118.57-fold increase compared to a 174.34-fold increase in unselected cells (Fig. 3E). Similar results were found for *SOX9* mRNA expression levels, a 56.89-fold increase and a 22.65-fold increase in unselected and Stro-4+ population, respectively (Fig. 3F).

Micromass culture in chondrogenic media showed a significant upregulation compared to basal conditions in this 3-D culture model. *COL2A1* showed a 9297.29-fold increase in unselected cells and a 4904.08-fold increase in Stro-4+ cells (Fig. 3D); *ACAN* showed a 3935.52-fold increase and a 2018.06–fold increase expression respectively (Fig. 3E). On the other hand, no significant increase in *SOX9* expression was observed (Fig. 3F).

## 7 Evaluation of bone formation using Stro-4+ oBMSCs in the bone defect/CAM model

**Fig. 4:**
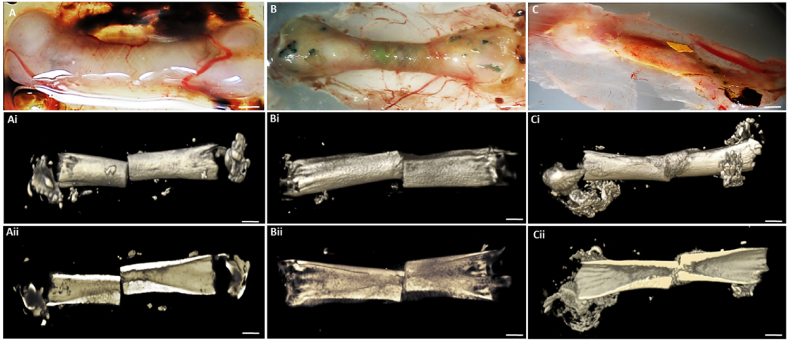
**CAM cultured chick femur defects with Stro-4+ mPCL ECM.** Femur with mPCL mesh only (blank scaffold) (A); Femur with mPCL and ECM (B); Femur with mPCL scaffold, ECM hydrogel seeded with Stro-4+ oBMSC (C); N = 3, Scale bar = 2 mm. Micro-Computed Tomography images of day 18 CAM femurs. **Stro-4+ mPCL scaffold ECM Hydrogel constructs.** Unseeded scaffold alone (Ai & Aii); Unseeded scaffold and blank ECM (Bi & Bii); mPCL scaffold and Stro-4+ Seeded ECM (Ci & Cii). No new bone growth was noted in blank scaffold. Areas of low opacity noted in the blank and blank ECM controls. New bone formation and bony bridging was observed in the Stro-4+ seeded ECM/mPCL scaffold group. N = 3, scale bar = 3 mm.

Bone formation was assessed using embryonic chick femur (E18) defect implanted with oBMSCs in a unique carrier system (mPCL + bECM) consisting of a proprietary mPCL microfiber scaffold and bECM hydrogel. Excellent integration was observed of the bone defect/microfiber scaffold/hydrogel/Stro-4+ (group 3)construct with the vascular rich CAM (Fig. 4A–C). In all three groups (control; blank mPCL scaffold), carrier (ECM) and carrier seeded with oBMSCs showed evidence of good integration with the CAM membrane (Fig. 4A–C) with marked soft tissue and blood vessel invasion into the implant. μCT evaluation in the control defect showed a clear boundary demarcation between femur segment ends with negligible signs of new bone growth (Fig. 4Ai & Aii). In the carrier group (mPCL scaffold and ECM hydrogel, unseeded) bone segments at either end of the defect could be delineated, and a modest degree of “softening” to the defect edges was evident, linked to early new bone tissue formation (Fig. 4Bi & Bii). In the Stro-4+ oBMSCs-mPCL scaffold (group 3), there was clear blood vessel and soft tissue ingrowth visible macroscopically. Blood vessel ingrowth was ubiquitous with numerous smaller vessels visible in comparison to the control and carrier alone groups. μCT analysis demonstrated the boundary defect region to be completely filled and bridged with new bone with a comprehensive volume of new bone formation in the structure of longitudinal bone columns and nodules under saggittal view. (Fig. 4Ci & Cii).

**Fig. 5:**
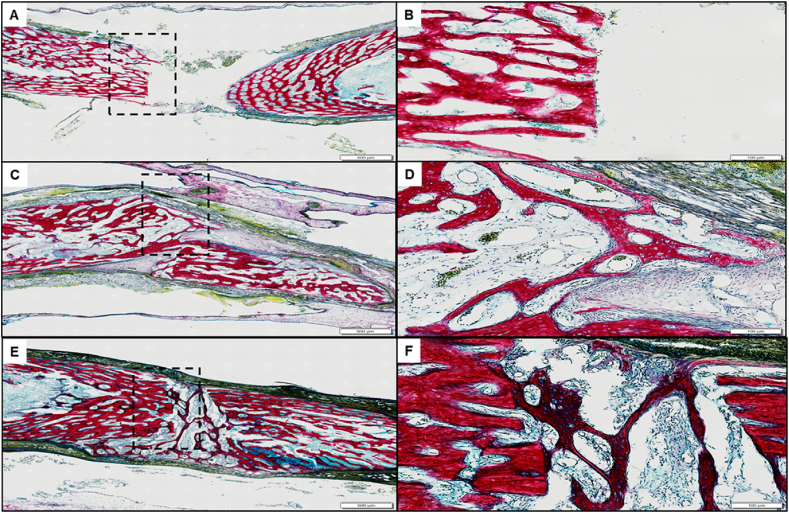
**CAM ECM-mPCL scaffold Alcian blue and Sirius Red histology.** A, B) Blank mPCL scaffold only. C, D) mPCL scaffold and unseeded ECM. E, F) mPCL scaffold with Stro-4+ oBMSCs seeded ECM. A, C, E scale bar = 500 μm. B, D, F scale bar = 100 μm. Defect boundary demarcated by the black dashed box (B, D, F). (For interpretation of the references to colour in this figure legend, the reader is referred to the Web version of this article.)

Histological sections stained for alcian blue/Sirius red demonstrated a clear demarcated perpendicular transection of the defect femur after the culture period in the femur defect with just the mPCL-scaffold alone, where the cut ends displayed no new bone outgrowth nor indications of cellular hypertrophy (Fig. 5A and B dashed black box). A mild periosteal reaction was observed as non-specific cellular infiltration into the periosteal region continuous with the peripheral cortical bone, indicating an obvious non-healing blank defect (Fig. 5A and B). With the addition of the ECM hydrogel into the mPCL scaffold encompassing the femur defect, the boundaries of the defect ends of the bone remained visible (Fig. 5C and D).

However, with the addition of Stro-4+ oBMSCs to the ECM partial bridging was observed originating from the central trabecular bone region, with new growth characterised by red-staining bone spicules containing entrapped hyperplastic cells in an enlarged peri-cellular space. The cells within the new bone spicules displayed negligible alcian blue staining. However, a thickened hyper-cellular connective tissue membrane appears to have invaded the defects space, with a degree of bony bridging and partial repair (Fig. 5E and F).

**Fig. 6:**
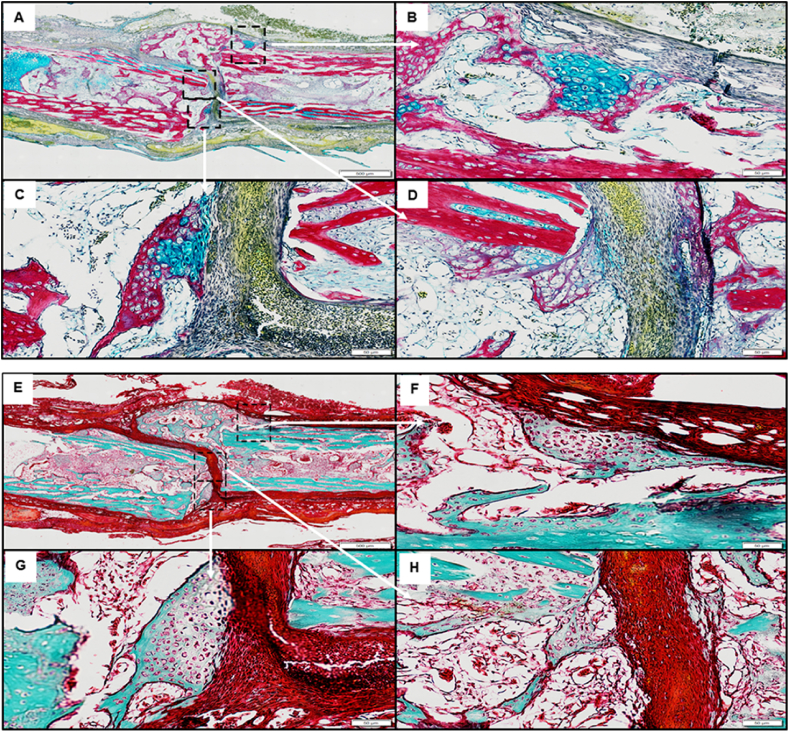
**Stro-4+ oBMSCs seeded ECM CAM/bone defect histology.** Sample overview of Alcian blue Sirius red histology section staining; 3 regions of interest demarcated by the dashed box (A). Cluster of proteoglycan producing cells (white arrow) associated with periosteum like membrane (B). Area of hypertrophic cells (white arrow) associated with invading fibrous sheet (*) (C). Fibrous cellular invasion (*) of defect invading cells appear extra-femoral in origin and have begun collagen and proteoglycan matrix deposition (D). Sample overview of mineralised and osteoid staining (Goldner's trichrome) (E–H); Area of hypertrophic cells showing central region of osteoid production (E). Highlighted regions of hypertrophic cells showing clear osteoid production (white arrows) (G, H), cells are associated with invading collagen, osteoid-like dense tissue (Black arrows). Scale bar A&E = 500 μm, B, C, D, F, G, H = 50 μm. (For interpretation of the references to colour in this figure legend, the reader is referred to the Web version of this article.)

Within the femur defect site containing the Stro-4+ cells encapsulated in the acellular mPCL scaffold-hydrogel the defect boundary could be identified by columns of bone, orientated parallel to the diaphyseal axis (Fig. 6A and B). Trabecular-like new bone growth, bridging the bone defect were observed by extensive Sirius red staining (Fig. 6C and D). Areas of new bone were observed consistent with periosteum-like material surrounding the femoral diaphysis. (Fig. 6F).

Interestingly, the trabecular-like bone appeared to have developed in a perpendicular orientation to the bone defect ends (Fig. 6E and F). Hypertrophic cell clusters within the newly formed bone were rich in proteoglycans (Alcian blue staining) (Fig. 6B and C) furthermore, seams of osteoid matrix were observed peripheral and centrally within the bridge defect site (Fig. 6E–H).

## 8 Evaluation of oBMSCs modulation of bone formation using bECM and mPCL scaffolds in an ovine tibial segmental defect

### 8.1 μCT

**Fig. 7:**
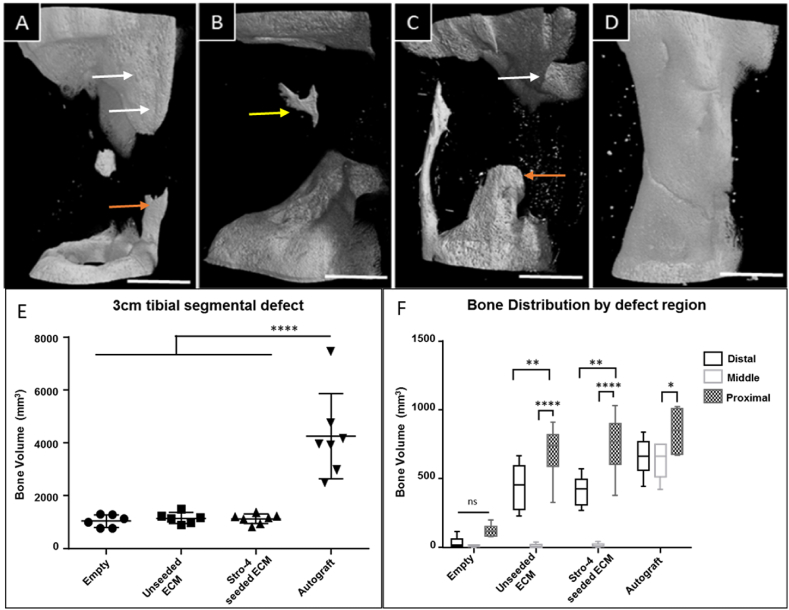
**Images of the highest bone volume from each experimental group.** Empty defect (A), unseeded ECM, region of new bone outgrowth originating from the distal medullary canal (B), Stro-4+ seeded ECM, new bone formation centrally within the defect unrelated to defect periphery or fixation plate (yellow arrow) (C), autograft (D). White arrows indicate bone growth associated with fixation implant, orientated uniformly on the cranio-medial aspect of the tibia. Bone growth along the neurovascular bundle was present in many samples across all groups, orange arrow. Scale bar = 1 cm**. Ovine segmental defects μCT analysis.** A statistically significant difference in bone volume was observed between autograft (n = 7) and blank (n = 6), unseeded ECM (n = 6) and Stro-4+ seeded ECM (n = 7) (E). There were no significant differences amongst other groups. Micro CT analysis of bone distribution by distal, middle and proximal tibial defect regions (F). (For interpretation of the references to colour in this figure legend, the reader is referred to the Web version of this article.)

Micro-CT analysis was performed on data from the current experiment and combined with historical control data from Ref. [32] 3-D renders were compiled for qualitative comparison and bone volume analysis performed for quantitative analysis. The growth profile within blank samples was characterised by a low total amount of new bone and the absence of bone bridging at the three-month time point. In all cases, a degree of ossification was seen along the fixation plate. The total amount of bone in the area was variable but clearly distinguished from bone growth originating from the defect boundaries indicating “new bone”. Spicules of bone were observed orientated along the line of the neurovascular bundle, unlike plate-related bone growth, although this was not always present. The degree of new bone originating from the bone medulla and cortical edges was minimal. New growth from these areas rarely projected into the defect more than 5 mm from the defect edges. In some cases, new bone was detected around the periphery of the defect, interpreted to be related to the mPCL scaffold. In no case was any new bone present in the centre of the defect in the blank samples (Fig. 7A–D).

Autograft samples displayed comprehensive bone bridging of the defect in all cases (Fig. 7D). There was a small variation in the diameter of the bone bridge, in only one sample was the diameter of new bone less than half of the intact cortices either side of the defect. Autograft samples also displayed large amounts of new bone around the plate - most evident on sample retrieval whereupon the plate was encased in new bone. The large volumes of new bone were readily distinguishable on micro-CT from un-operated bone evidenced by; i) a change in cortical pattern internally and ii) a visually distinctive surface pattern externally.

Unseeded and seeded test samples were, typically, visually indistinguishable from empty control samples. There were indications of bone growth which did not match that observed in empty controls. These changes related to prominent bone outgrowth from the medullary canal (unrelated to intact cortices), low-density irregular spiral patterned bone outgrowth from proximal and distal defect ends, and in one case, a consolidated high-density bone fragment located centrally within the defect as highlighted in Fig. 7A–D.

Autograft samples contained significantly more bone, (4250.63 mm^3^, SD = 1485.57) than mPCL (blank) (1045.29 mm^3^, SD = 219.68) mPCL + ECM (unseeded; group 2) (1152.58 mm^3^, SD = 191.95) and mPCL + ECM + oBMSCss (cStro-4^+ve^ seeded bECM; group 3) (1127.95 mm^3^, SD = 166.44) groups, (Fig. 7E). There was no significant difference in the volume of bone measured in blank, unseeded and seeded ECM groups. New bone growth (bone volume) distribution was analysed according to the region with the defect. The total defect was sub-divided into proximal, middle and distal portions to investigate whether a gravitational effect or displacement of hydrogel had occurred and if so, whether the effect translated into a variation in the location of new bone within the defect. In all groups, the largest proportion of bone was located in the proximal segment. In seeded and unseeded groups, bone volume in the proximal segment was significantly larger than in the middle and distal portions. The proximal region of autograft samples was significantly enhanced in the middle sections only. A distinct feature of bone distribution in blank, unseeded and seeded groups was the near complete absence of bone measured in the middle portions. In the Blank samples, there were no differences between the three sub-regions (Fig. 7F).

**Fig. 8:**
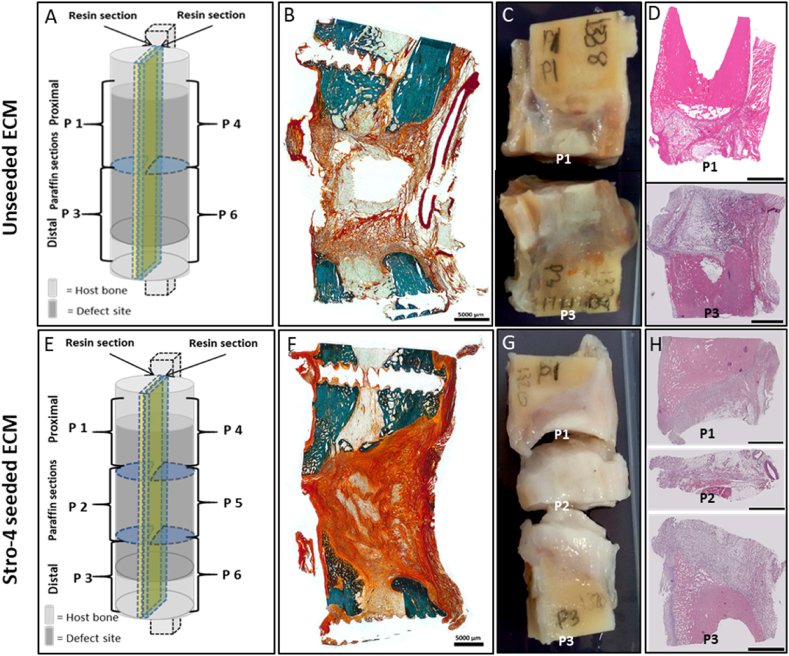
**Histology images of the tibial defect and ECM hydrogel mPCL scaffold groups**: Unseeded ECM (A–D); Stro-4+ seeded ECM (E–H). Schematic diagram of the tibial defect samples, depicting regions of the histological sections selected for analysis in unseeded and Stro-4 seeded ECM samples (A & E); Goldner's trichrome staining of tibial sections (B & F); Macroscopic images of the tibial sections obtained from either the proximal or distal regions of the tibial defect (C & G); Haematoxylin and Eosin stained sections of the proximal and distal regions of the tibial defect (D, H). Scale bar 5000 μm.

In the mPCL and unseeded ECM samples (Fig. 8A–D), haematoxylin and eosin staining (Fig. 8D) indicated poorly aligned tissue with minimal cellular content. Proximal and distal segments demonstrated highly cellular marrow cavity components and orientated matrix co-localised with innate bone. Stro-4^+ve^ seeded samples (Fig. 8E–H), showed a similar morphology to the unseeded sample under H & E staining (Fig. 8H). The defect material was characterised by a wash of non-distinct (pink/purple) eosinophilic material and a low level of nuclear basophilic material. Central areas lacking staining may represent regions of dissolved lipids. Overall, the histological analysis within these samples indicated repair consistent with fibrous non-union. In the unseeded ECM samples, Goldner's Trichrome (Fig. 8B), the proximal and distal tibial segments of innate bone stained a vibrant blue/green. A small amount of open structured osteoid was visible sprouting from the bony defect edges. The centre of the defect was devoid of nucleated tissue, and an empty void was seen on the section after processing. The central void was surrounded by a loose network of connective tissue staining orange-red. The region of fibrous tissue was poorly cellularised, with a randomly deposited matrix containing pockets of adipose like tissue. In the Stro-4 seeded ECM samples, Goldner's trichrome (Fig. 8F) staining demonstrated the presence of mineralised bone on the proximal and distal defect boundaries was observed. The defect substance was characterised by dense bundles of red/orange staining collagen fibres and cytoplasm. The fibre distribution was amorphous and disorganised and contained a discrete amount of adipose like material. The fibre density in the seeded group was greater than that in the unseeded with a majority of the defect substance intensely staining, with only small areas lacking evidence of collagen (Fig. 8B and F).

## 9 Discussion

In the current study, we have characterised and studied Stro-4+ oBMSCs in comparison to unselected oBMSC populations and examined their musculoskeletal regenerative potential *in vitro* and in small and large *in vivo* models using bovine derived ECM hydrogels and a biocompatible electro-written PCL. Our *in vitro* studies demonstrated oBMSC Stro-4 expression declined with subsequent passaging in monolayer culture with negligible detection at passage 4. This accounts for the slow initial PDT demonstrated during the post isolation P0 phase, both unselected and Stro-4+ populations followed a similar cellular proliferation profile. Ovine BMSCs expressed high levels of MSCs associated markers including CD29, CD44, CD90, CD166 and Stro-4 with a negligible expression of the endothelial associated adhesion marker CD31 and the haematopoietic markers CD45 and CD34 in keeping with our previous reports [7,12]. The persistence of Stro-4 in oBMSCs has not yet been documented but is in keeping with the pattern noted in human Stro-1 positive BMMNCs [36]. Growth rates, measured by population doubling time, in unselected and selected cells were comparable between populations at each passage between P1 and P5 and was comparable to results demonstrated by others in sheep [9,20] and humans [20,37,38]. The CFU-F capacity of whole, lymphoprep separated BMMNCs from ovine bone marrow presented with a morphological and colony-forming potential comparable to findings by Bruder and Gronthos (above) and similar to CFU-F data reported in human unselected MSCs [[38], [39], [40]]. Stro-4 CFU-F was documented by Gronthos et al.*,* 2009 [12] on freshly sorted P0 cells, however, CFU-F formation with continued passage was not recorded. The results in this study obtained on growth rate and colony-forming capacity of ovine unselected BMSCs, compared favourably with the literature and showed a close homology in the performance of BMSCs between humans and sheep.

A defined component of an MSC is its ability to differentiate into skeletal elements. *In vitro* differentiation on 2-D monolayer culture under defined conditions results in a demonstrable phenotypic change towards osteogenic, chondrogenic and adipogenic phenotypes. In monolayer cultures, both unselected and Stro-4+ cells, under chondrogenic culture conditions, significantly enhanced COL2A1, ACAN and SOX9 gene expression showing a high degree of differentiation towards a chondrogenic phenotype. A similar expression pattern was established in the micromass experiments, although it appeared that micromass culture was less responsive to further stimulation by chondrogenic media. When examining chondrogenic matrix deposition by alcian blue staining, Stro-4+ cells at early passage significantly outperformed unselected cells. Discordantly, upregulation in gene expression across both cell groups was observed without any clear improvement in chondrogenic potential between selected or unselected groups. The current studies indicate a propensity for ovine cells to differentiate more readily towards a chondrogenic lineage rather than an osteogenic or adipogenic phenotype. This is in contrast to the predominance of Stro-1^+ve^ cells from adult human donors to differentiate along an osteogenic lineage [36]. Human foetal chondrocytes and MSCs are known to share common MSC markers and are known to express higher levels of Stro-1 than an adult cell population [41]. The inclination of ovine selected, and to a lesser degree, unselected bone marrow stromal cells towards chondrogenesis may imply that these cell populations are able to retain an earlier differentiation lineage phenotype. Work by Gotherstrom et al., 2005 [42] demonstrated that the gene expression of human adult and foetal MSCs varied with foetal MSCs expressing a less lineage-specific phenotype. One explanation for the difference in differentiation behaviour between human Stro-1 and ovine Stro-4 may include a more primitive phenotype in ovine Stro-4 cells or a propensity for chondrogenic differentiation facilitating endochondral ossification. Work comparing foetal and mesenchymal stem gene expression would be needed prior to drawing more assertive conclusions.

The significant osteogenic advantage seen in alizarin red staining of early and late passage Stro-4+ cells over unselected was not reflected in gene expression analysis. Neither *OCN* nor *COL1A1* expression were significantly different in basal and osteogenic conditions in either cell group. Osteogenic gene expression did correlate with a positive difference in alizarin red staining noted in osteogenic media of early passage cells. A variable temporal expression of *COL1A1* and *OCN* in human MSCs *in vitro* has been previously demonstrated [43] however, in our studies gene expression was only performed on day 21 cultures of ovine Stro-4+ selected and unselected cell populations and may not have captured peak *COL1A1* and *OCN* expression. Surprisingly, upregulation of the adipogenic marker *PPARG* was significantly higher in the Stro-4+ selected population in both basal and adipogenic conditions.

The bone and cartilage forming ability of Stro-4+ oBMSCs were assessed in a high-throughput orthotopic chick CAM femur assay using a novel mPCL scaffold ECM hydrogel system. Previously, the *in-vivo* bone and cartilage forming capacity of ovine MSCs has been shown on HA/TCP and Gelfoam models specific to lineage differentiation [7] and that the incorporation of skeletal populations into ECM hydrogels has been demonstrated to enhance bone and cartilage regeneration *in-vivo* [[44], [45], [46]]. We demonstrated using a novel mPCL scaffold ECM hydrogel system that in the CAM model (with its proliferative vasculature) unseeded hydrogels demonstrated a limited degree of osteoinduction showing a hypertrophic periosteal reaction and partial bone bridging. Significant new bone formation was observed in the Stro-4+ oBMSCs seeded scaffold, which was qualitatively superior to ECM hydrogel alone when assessed for evidence for orthotopic osteochondral ossification. In addition, the external texture of new bone was pitted in appearance, the significance of which is unknown but may relate to blood vessel invasion or osteoclastic bone remodelling. We found that staining concurrently for proteoglycan and calcium aggregation indicated the presence of chondrogenesis centrally, with osteoblast differentiation and mineralisation peripherally validating a claim for endochondral ossification. New bone formation appeared orientated along invading fibrous tissue and vascular structures and clear differentiation between innate bone and newly formed bone. Invading fibrous structures appear intimate and consistent with femoral periosteum, which leads to the presupposition that new bone is highly dependent on the nature of this invading tissue and its interaction with ECM and Stro-4 elements. Bone cells release a number of mitogenic growth factors including VEGF, IGF, EGF and TGFβ-1 and their paracrine effects on periosteum resident skeletal cells enhance wound healing *in vivo* [47]. Periosteum derived MSCs are multipotent and responsive to both osteogenic and chondrogenic differentiation *in-vivo* [48], periosteum and bone marrow derived MSCs have been shown to upregulate type II collagen production in response to TGF β1 [49]. Furthermore, MSCs are responsive to chondrogenic and osteogenic factors and, MSC exposure to chondroblast conditioned media enhances bone and cartilage formation *in-vivo* [50].

Paracrine effects may be inferred by the degree of enhanced periosteal hypertrophy and association of new bone directly with none-bone tissues seen in the seeded compared to the unseeded group. Stro-4+ oBMSCs displayed a chondrogenic phenotype even under basal conditions leading to a hypothesis that the priming influence of Stro-4 enhanced the chondrogenic and osteogenic effect on periosteal skeletal precursors when implanted *ex vivo* on to the CAM. The extent to which Stro-4 contribute directly to new tissue formation through proliferation and differentiation cannot be determined despite *in vitro* determination of growth kinetics as no form of tracking was employed in the seeded population. Furthermore, the response of Stro-4+ cells in comparison to, for example, Stro-1^+ve^ cells between ovine and human populations is unknown.

The development of novel tissue engineering therapies requires a comprehensive *in vivo* review in animal models to generate clinically relevant datasets. Orthopaedic research, in particular, requires animal models of a comparative size, mass, biomechanics and physiology to the human patient. Where rodent models are easily accessible and less expensive they have differing bone morphology to humans and are too small to critically assess biodegradable repair constructs in bone particularly in combination with fixation or critical-sized fracture repair. Hence, sheep have emerged as a well-accepted model expanding our understanding of species bone structure, anatomy, metabolism and physiology and therefore fulfilling many of the favourable selection criteria in translational medicine. Understanding of the cellular biology within any model is an essential component of regenerative medicine, in particular, the cells which participate in self-renewal and repair. Although much is known about human MSC populations, debate remains surrounding the origin, phenotype, tissue location and function. Previous work by Gronthos and others has opened up our understanding of ovine MSCs both *in vitro* and *in vivo* [7,10,51] but relatively little is known concerning selected ovine BMSC sub-populations. Thus, the current studies build on our previous work on ovine bone marrow derived cell populations using autologous and allogenic cells in cell-seeded scaffolds and unloaded scaffolds with autologous bone grafts as a control group [11] with bone formation assessed 12 weeks post-surgery. We observed no significant differences in bone formation between the autologous and allogenic groups, in those studies, while we observed enhanced bone formation using cells. Critically biomechanical analysis indicated no significant differences between the cell groups and the unloaded scaffolds. In addition, using our standardised 3-cm, critical-sized tibial ovine defect model there was improved reparative capacity on delayed injection of allogeneic skeletal cell populations to a defect site [52]. We have also reported the reparative capacity of such a tissue engineered construct was less than for autograft and/or a BMP-7 loaded scaffolds [53]. We have also published on a study determining whether osteoblasts (OBs) isolated from the axial skeleton (tOBs) differ from OBs of the orofacial skeleton (mOBs) given the different embryological origins of the bones and their capacity for bone regeneration in a CSD in sheep [54]. We found no significant differences following biomechanical, microCT and histological analysis of the bone regeneration potential of tOBs and mOBs in our *in vitro* study, as well as in the bone regeneration potential of different cell types *in vivo*. These studies provided the backdrop to the current studies that have centred on the analysis of Stro-4 enriched ovine skeletal populations for bone reparation and we have not re-examined again non-selected/unsorted bone marrow stromal cells given our existing published data and, importantly, to address the important principles surrounding 3Rs animal experimentation of replacement, refinement and reduction.

The use of large animal models in pre-clinical translation continues to present a necessary yet challenging step in the development of novel therapeutics. However, testing these new therapies from the bench to large *in vivo* animal models is not without out its complications particularly using a multifaceted approach as described in the current study. Thus, cell scale up using Stro-4+ oBMSCs, matrix hydrogel and melt electro-written micro-fibre mPCL scaffold generation provided challenges for the sheep tibial defect model. Our results demonstrated a non-significant level of bone forming activity compared to controls. This may be due to a number of components including i) the length of the study, insufficient to generate new bone or, with the hydrogel production, ii) nutrient supply and modulated Stro-4 survival over time and, iii) Limited regeneration of the vasculature with the formation of new bone, in such a large ovine defect, may all be contributory factors in the limited regeneration of such a large osseous defect. Although, the current studies do not support a strong role for Stro-4 cells in the support of bone formation, it cannot be excluded that an insufficient number of cells survived across the implantation time frame to support bone reparation. The manufacture of large volumes of ECM gel (100 fold greater volumes than required for CAM models), development of a homogenous buffered gel proved challenging and may have led to discrete regions of variable pH that were difficult to neutralise, subsequently affecting skeletal cell activity.

The central role of the mPCL scaffold in the current study was to localise the bECM hydrogel, permit nutrient diffusion and facilitate vascular penetration. Melt electro-written micro-fibre mPCL scaffolds have been produced and characterised *in vitro* [29,55,56] and utilised successfully *in vivo* [57,58]. Previous applications documented the successful use of a mPCL scaffold fabricated via fused deposition modelling technology to improve the localisation of incorporated autograft and platelet rich plasma in combination with BMP-7 [59]. Solution electrospinning technology was also used to produce fibrous PCL scaffolds, which localised a cross-linked alginate hydrogel containing BMP-2 [60]. Autograft was an effective positive control, with significant bridging of the defect observed. The use of the mPCL scaffold membrane on a modest scale, as demonstrated in the successful CAM model, corroborated applications previously reported [55,59,60]. In comparison, the use of mPCL scaffold for gel localisation in a large animal critical-sized defect may not have successfully localised the biomaterial for a duration sufficient to stimulate osteogenesis. It is not known whether a failure to localise in an ambulatory large animal was a contributing factor, gel at the time of application in surgery was well contained and the construct sound.

Interestingly, in the current studies, Stro-4 oBMSCs displayed a propensity to differentiate along the chondrogenic lineage and suggests an impact on osteogenesis within the implanted Stro-4+ cells/hydrogel. In addition, the lack of cell proliferation observed and minimal vascular response, in marked contrast to the chick CAM vascular-rich environment, may infer that to orchestrate a reparative response in a large defect model, the rapid development of the vasculature to the site, as indicated above, is critical for success.

The question of autologous, allogenic and xenogenic material and potential deleterious effects on repair warrants consideration. An inability of bovine origin ECM hydrogel to stimulate an osteogenic effect could relate to the xenogenic nature of the primary tissue. In an acute inflammatory response, the release of pro-inflammatory cytokines such as IL-1, IL-2 and TNFα could modulate the pro-osteogenic signalling derived from matrix bound growth factors such as the TGF-β and BMP-2. Indeed, the presence of a fibrous cellular capsule around the cell-seeded and unseeded groups would support an inflammatory interaction. The use of cancellous bovine xenograft has been reported clinically in tibial fracture repair [61], maxillofacial reconstruction [62] and reconstructive foot surgery [63]. Although widely used and with reported improved efficacy over other common bone graft substitutes experimentally [64], the efficacy and suitability of xenograft in orthopaedics remains an area of considerable debate [65].

A review of ECM xenograft materials failed to identify a reliable description of the human immune response to xenogenic ECM grafts [66]. Importantly, when examining a xenograft related immunogenic response in an ovine model, work by Katz et al., [67] showed comparable osteogenesis between decellularised xenograft and allograft. The immunogenicity of connective tissue grafts has been linked to the cellular component of the graft tissues. Decellularisation and delivery of an acellular graft material has been shown to reduce and even remove the immune response of the host to donor material [65]. Using hydrogels as a cell delivery vehicle has been implicated in generating an enhanced immune response, this was primarily linked to the adsorption of cell proteins and presentation to host immunocytes [68].

## 10 Conclusions

The current studies have documented the *in vitro* and *in vivo* phenotype and function of an enriched skeletal stem cell population selected using the monoclonal IgG Stro-4 antibody and efficacy for bone formation using a novel mPCL scaffold ECM hydrogel system. oBMSCs demonstrated evidence of an *in vitro* growth profile comparable with human skeletal stem cells. A clear preference for ovine unselected and Stro-4+ oBMSCs to differentiate along the chondrogenic lineage was observed, an observation noted in the CAM chick femur defect model where the vascularised rich environment enhanced the bone repair when Stro-4+ cells in combination with bone-derived ECM hydrogels were applied. In a large bone defect model, the propensity of Stro-4+ cells to align to a chondrogenic lineage may have delayed the onset of the fracture repair. In addition, the deficiency of a functional blood supply to the ovine defect model would have certainly reduce the regenerative capacity of the composite-Stro-4+ cells, ECM and sleeve. The current studies highlight discrete differences between Stro-1 and Stro-4 skeletal populations with an innate chondrogenic lineage preference in comparison to published observations for osteogenesis in Stro-1 enriched adult human cells. The results validate the use of Stro-4+ skeletal populations and pave the way for *in vivo* evaluation of skeletal populations in tissue engineering and regenerative medicine, but other mitigating factors such as mechanics, vascularisation need to be incorporated to large scale up fracture models for these skeletal progenitor cells function at the optimum conditions. Finally, the current studies indicate the issues around translation from *in vitro* and small animal models to predict surgically-relevant efficacy and the challenges and unreliability that can be observed. It is clear, in the context of large scale skeletal tissue engineering, a targeted nature is required to meet the important issues around Reduce, Refine and Replacement for animal use in translational research. Ongoing studies are focussed on the translational potential of Stro-4+ oBMSCs and our scaffolds in a translational model of bone tissue engineering examining cell delivery, cell concentration and the potential of osteogenic pre-conditioning prior to implantation for clinical translation. The development of skeletal cell selection strategies in combination with deliverable composites and hydrogels in preclinical translational models auger well for the generation of hard and soft tissues for the ageing population.

## CRediT authorship contribution statement

**C. Black:** Conceptualization, Methodology, Data curation, Writing - original draft, Writing - review & editing. **J.M. Kanczler:** Methodology, Writing - original draft, Writing - review & editing. **M.C. de Andrés:** Methodology, Data curation, Writing - review & editing. **L.J. White:** Resources, Writing - review & editing. **F.M. Savi:** Methodology, Data curation, Writing - review & editing. **O. Bas:** Methodology, Writing - review & editing. **S. Saifzadeh:** Methodology. **J. Henkel:** Methodology, Writing - review & editing. **A. Zannettino:** Resources, Methodology, Writing - review & editing. **S. Gronthos:** Resources, Methodology, Writing - review & editing. **M.A. Woodruff:** Resources, Data curation, Methodology, Writing - review & editing. **D.W. Hutmacher:** Resources, Conceptualization, Project administration, Writing - review & editing. **R.O.C. Oreffo:** Conceptualization, Formal analysis, Funding acquisition, Project administration, Resources, Supervision, Writing - original draft, Writing - review & editing.

## Declaration of competing interests

The authors declare that they have no known competing financial interests or personal relationships that could have appeared to influence the work reported in this paper.

## Data Availability

The raw/processed data required to reproduce these findings cannot be shared at this time as the data also forms part of an ongoing study.

## Appendix A Supplementary data

Multimedia component 2 Multimedia component 2

Supplementary Figure 1 CellProfiler quantification of Alizarin Red Staining: Alizarin Red quantification of P2 Ovine Stro-4+ cells. A) Basal condition with DAPI staining inset. B) Osteogenic media with DAPI inset. C) Transformed image of basal conditions. D) Transformed image of osteogenic conditions. Scale bar 500 μm.. Supplementary Figure 1

Supplementary Figure 2 CAM experimental set-up including cell culture ECM gel and mPCL scaffold preparation. A) Stro-4+ oBMSCs at P0 in culture, scale bar 100 μm B) Cell culture hood containing all elements of set-up C) 2× Magnification of melt electro-written scaffold prior to implantation, scale bar 1 mm D) mPCL scaffold and explanted femurs prior to construct formation. E) Femur scaffold constructs. F) Implantation onto CAM. G) Explanted femur and scaffold with integrated CAM at day 8 of CAM culture, scale bar 5 mm. Supplementary Figure 2

Supplementary Figure 3 **Melt electrowriting process and fabricated tubular medical-grade polycaprolactone (mPCL) scaffolds for sheep tibial defect.** (A) The tubular printing configuration of melt electrowriting device which consists of a print head and rotational collector. The image also shows the deposition of the generated jet of molten mPCL. B) Representative image of the fabricated tubular mPCL scaffold (~6 cm in length, ~2 cm in diameter) with (C) its scanning electron microscopy micrograph. Supplementary Figure 3

Supplementary Figure 4 **Scaffold and bECM application**. Completed osteotomy and defect. A) Defect region created, proximal and distal tibial portions without fixation. B) Application of bECM scaffold onto proximal tibial segment. C) Syringe with 8 mL of bECM, D) Scaffold applied and secured by suture and plate, proximal segment. E) bECM injected into scaffold lumen. F) Completed defect and construct *in situ*. Supplementary Figure 4

Supplementary Table 1 Primer sequences for qRT-PCR Supplementary Table 1

Supplementary Table 2 Experimental groups for the ovine tibial defect study Supplementary Table 2
