# Supplementary figures and images for: Characterisation and evaluation of the regenerative capacity of Stro-4+ enriched bone marrow mesenchymal stromal cells using bovine extracellular matrix hydrogel and a novel biocompatible melt electro-written medical-grade polycaprolactone scaffold
Source: Biomaterials. 2020 Jul;247:119998. doi: 10.1016/j.biomaterials.2020.119998 (PMC7184676; doi:10.1016/j.biomaterials.2020.119998)

## Slide 1
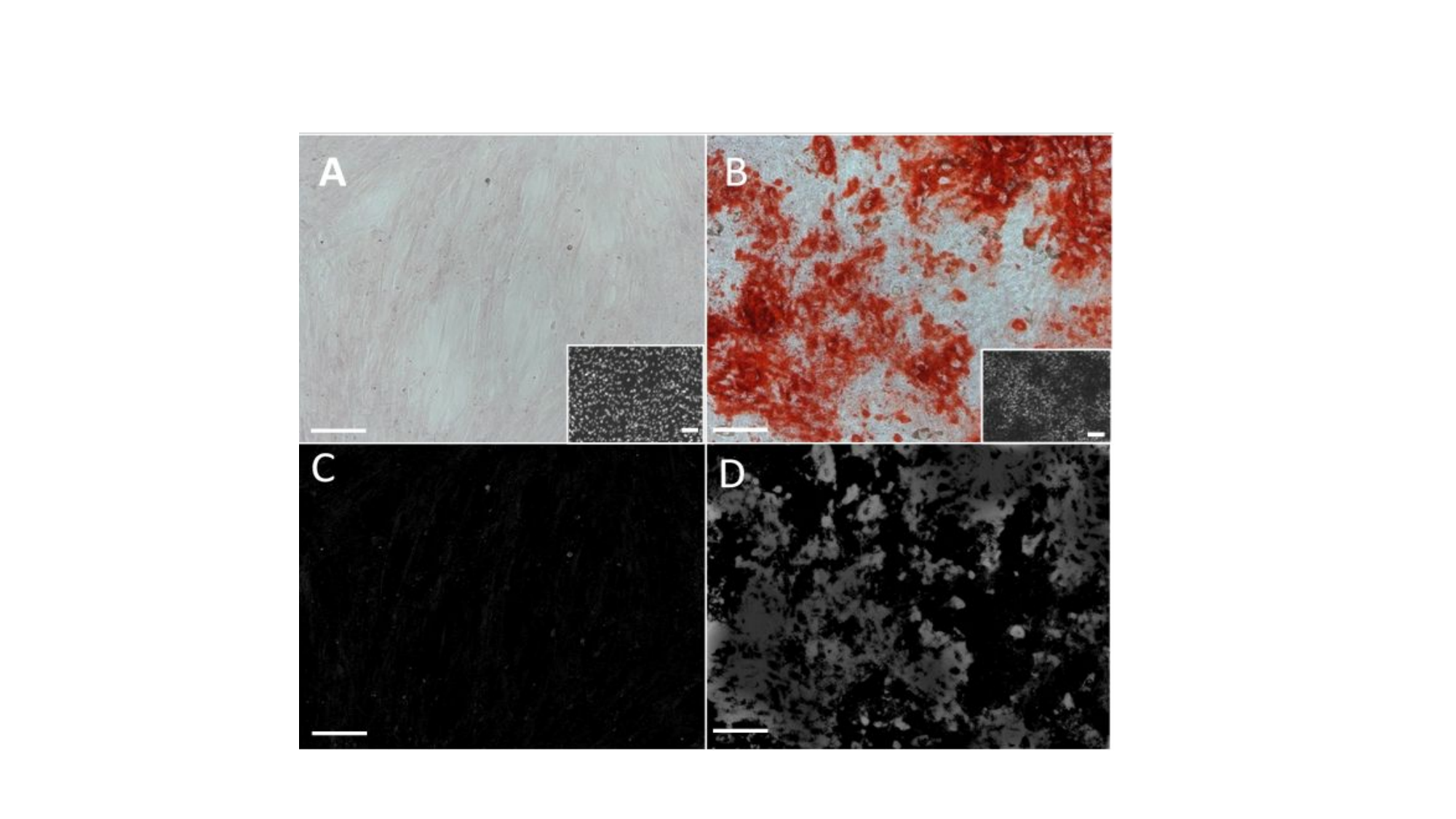

Supplement: Supplementary Figure 1 — CellProfiler quantification of Alizarin Red Staining: Alizarin Red quantification of P2 Ovine Stro-4+ cells. A) Basal condition with DAPI staining inset. B) Osteogenic media with DAPI inset. C) Transformed image of basal conditions. D) Transformed image of osteogenic conditions. Scale bar 500 μm.. [file mmc3.pptx]

## Slide 1
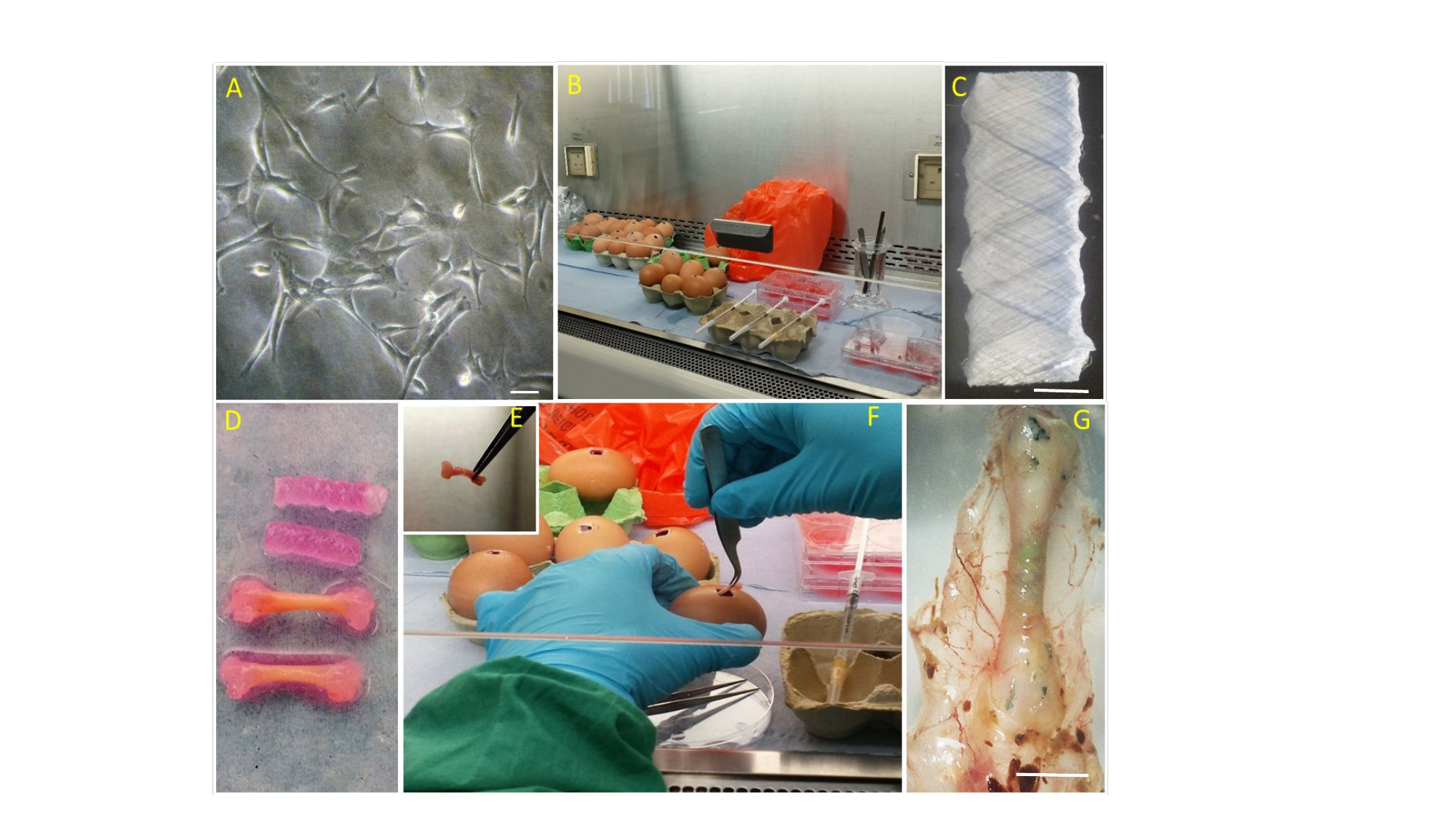

Supplement: Supplementary Figure 2 — CAM experimental set-up including cell culture ECM gel and mPCL scaffold preparation. A) Stro-4+ oBMSCs at P0 in culture, scale bar 100 μm B) Cell culture hood containing all elements of set-up C) 2× Magnification of melt electro-written scaffold prior to implantation, scale bar 1 mm D) mPCL scaffold and explanted femurs prior to construct formation. E) Femur scaffold constructs. F) Implantation onto CAM. G) Explanted femur and scaffold with integrated CAM at day 8 of CAM culture, scale bar 5 mm. [file mmc4.pptx]

## Slide 1
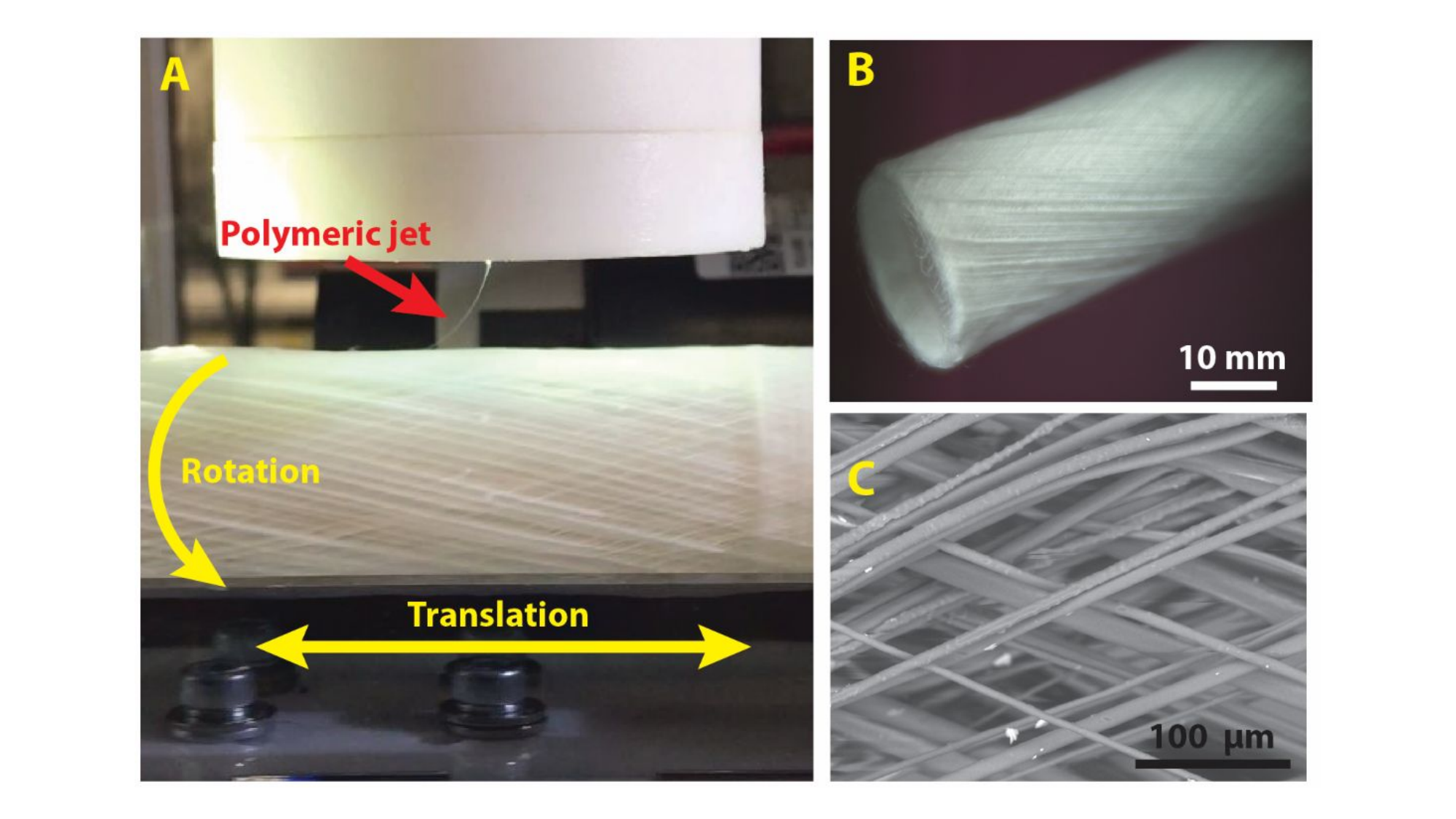

Supplement: Supplementary Figure 3 — Melt electrowriting process and fabricated tubular medical-grade polycaprolactone (mPCL) scaffolds for sheep tibial defect. (A) The tubular printing configuration of melt electrowriting device which consists of a print head and rotational collector. The image also shows the deposition of the generated jet of molten mPCL. B) Representative image of the fabricated tubular mPCL scaffold (~6 cm in length, ~2 cm in diameter) with (C) its scanning electron microscopy micrograph. [file mmc5.pptx]

## Slide 1
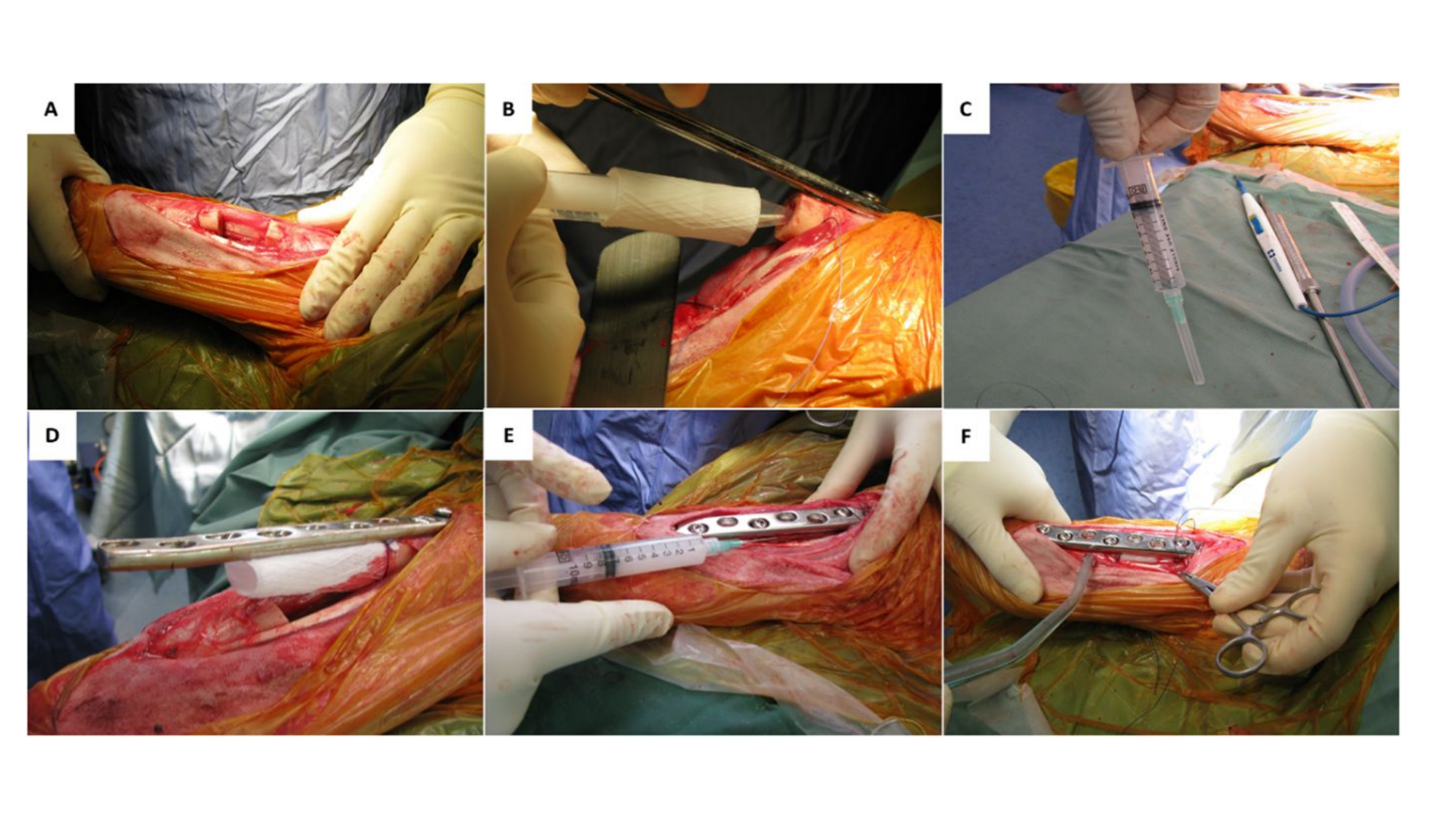

Supplement: Supplementary Figure 4 — Scaffold and bECM application. Completed osteotomy and defect. A) Defect region created, proximal and distal tibial portions without fixation. B) Application of bECM scaffold onto proximal tibial segment. C) Syringe with 8 mL of bECM, D) Scaffold applied and secured by suture and plate, proximal segment. E) bECM injected into scaffold lumen. F) Completed defect and construct in situ. [file mmc6.pptx]
