# Supplementary material for: Characterisation and evaluation of the regenerative capacity of Stro-4+ enriched bone marrow mesenchymal stromal cells using bovine extracellular matrix hydrogel and a novel biocompatible melt electro-written medical-grade polycaprolactone scaffold
Source: Biomaterials. 2020 Jul;247:119998. doi: 10.1016/j.biomaterials.2020.119998 (PMC7184676; doi:10.1016/j.biomaterials.2020.119998)
Supplement: Supplementary Table 1 — Primer sequences for qRT-PCR [file mmc7.pptx]

## Slide 1
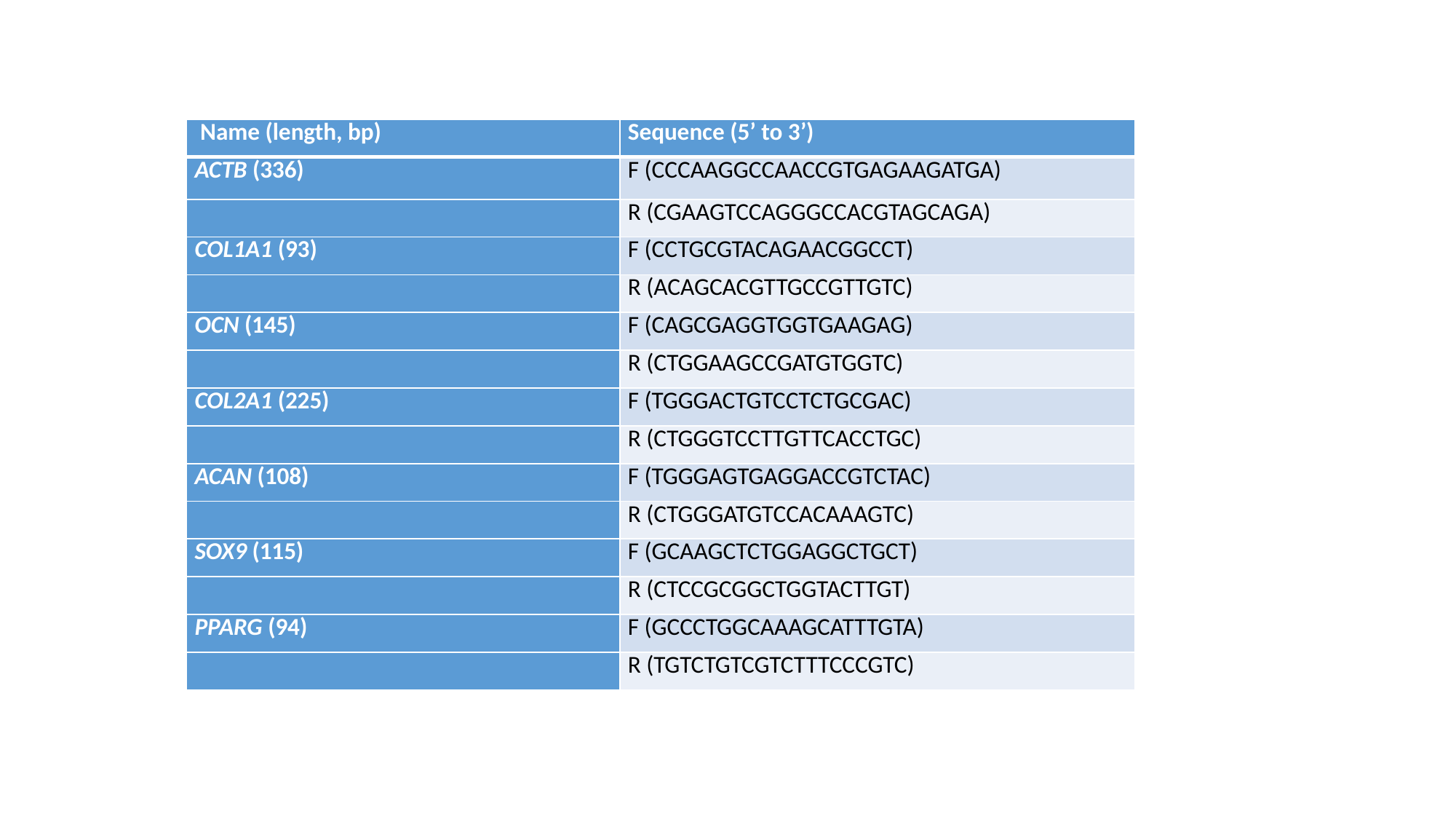

| Name (length, bp) | Sequence (5’ to 3’) |
| --- | --- |
| ACTB (336) | F (CCCAAGGCCAACCGTGAGAAGATGA) |
| | R (CGAAGTCCAGGGCCACGTAGCAGA) |
| COL1A1 (93) | F (CCTGCGTACAGAACGGCCT) |
| | R (ACAGCACGTTGCCGTTGTC) |
| OCN (145) | F (CAGCGAGGTGGTGAAGAG) |
| | R (CTGGAAGCCGATGTGGTC) |
| COL2A1 (225) | F (TGGGACTGTCCTCTGCGAC) |
| | R (CTGGGTCCTTGTTCACCTGC) |
| ACAN (108) | F (TGGGAGTGAGGACCGTCTAC) |
| | R (CTGGGATGTCCACAAAGTC) |
| SOX9 (115) | F (GCAAGCTCTGGAGGCTGCT) |
| | R (CTCCGCGGCTGGTACTTGT) |
| PPARG (94) | F (GCCCTGGCAAAGCATTTGTA) |
| | R (TGTCTGTCGTCTTTCCCGTC) |
