# Supplementary material for: Characterisation and evaluation of the regenerative capacity of Stro-4+ enriched bone marrow mesenchymal stromal cells using bovine extracellular matrix hydrogel and a novel biocompatible melt electro-written medical-grade polycaprolactone scaffold
Source: Biomaterials. 2020 Jul;247:119998. doi: 10.1016/j.biomaterials.2020.119998 (PMC7184676; doi:10.1016/j.biomaterials.2020.119998)
Supplement: Supplementary Table 2 — Experimental groups for the ovine tibial defect study [file mmc8.pptx]

## Slide 1
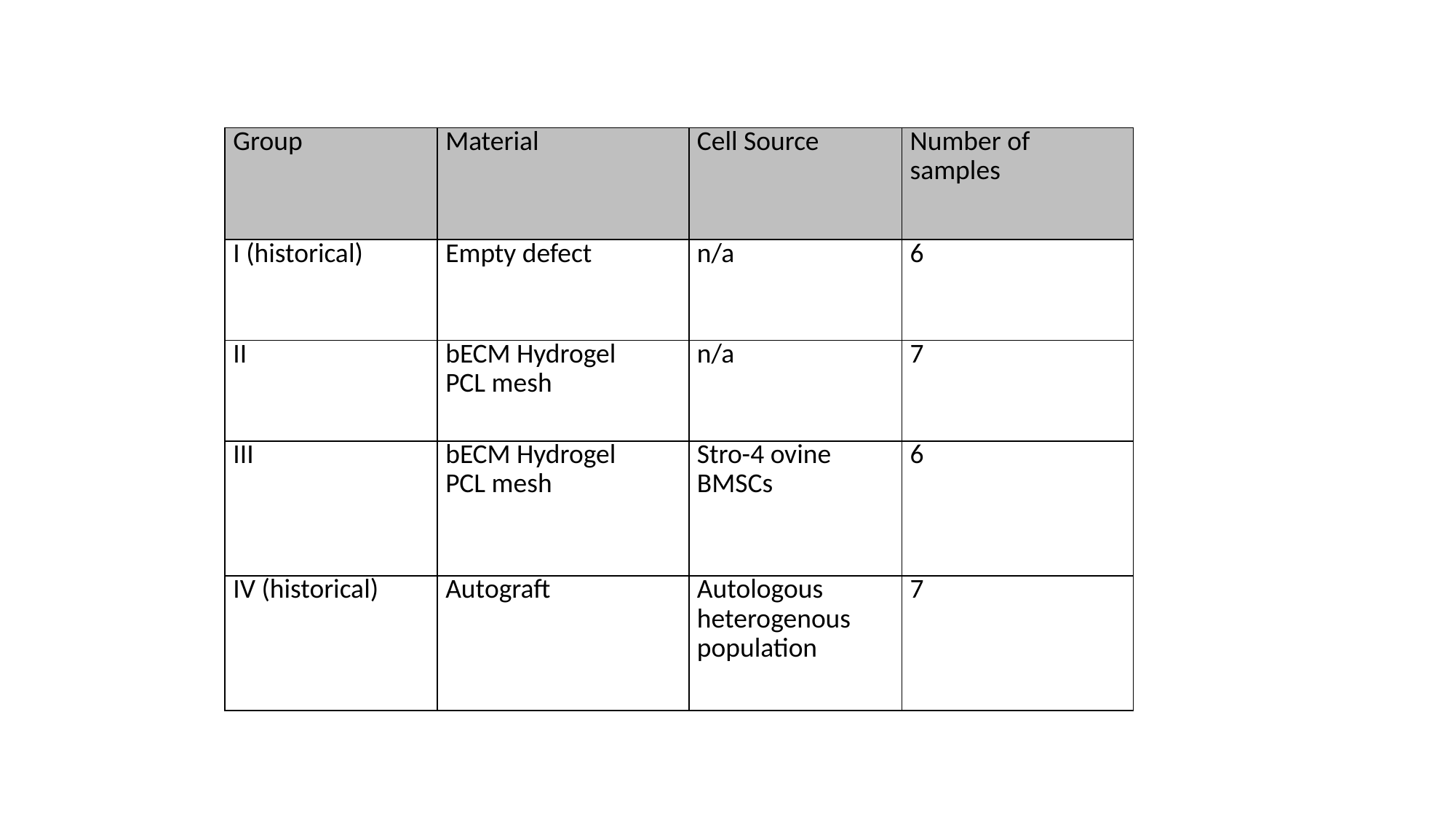

| Group | Material | Cell Source | Number of samples |
| --- | --- | --- | --- |
| I (historical) | Empty defect | n/a | 6 |
| II | bECM Hydrogel PCL mesh | n/a | 7 |
| III | bECM Hydrogel PCL mesh | Stro-4 ovine BMSCs | 6 |
| IV (historical) | Autograft | Autologous heterogenous population | 7 |
